# Immunoinformatics Design of a Multiepitope Vaccine (MEV) Targeting *Streptococcus mutans*: A Novel Computational Approach

**DOI:** 10.3390/pathogens13100916

**Published:** 2024-10-21

**Authors:** Romen Singh Naorem, Bandana Devi Pangabam, Sudipta Sankar Bora, Csaba Fekete, Anju Barhai Teli

**Affiliations:** 1 Multidisciplinary Research Unit, Jorhat Medical College and Hospital, Jorhat 785001, India; romen86@gamma.ttk.pte.hu (R.S.N.); sudip.asm@gmail.com (S.S.B.); 2 Department of Molecular Biology and Microbiology, University of Pecs, Ifusag utja. 6, 7624 Pecs, Hungary; bandanapangabam@gmail.com; 3 Department of Biochemistry, Jorhat Medical College and Hospital, Jorhat 785001, India

**Keywords:** *Streptococcus mutans*, dental caries, multiepitope vaccine, molecular docking simulation, molecular dynamic simulation, immunoinformatics

## Abstract

Dental caries, a persistent oral health challenge primarily linked to *Streptococcus mutans*, extends its implications beyond dental decay, affecting over 4 billion individuals globally. Despite its historical association with childhood, dental caries often persists into adulthood with prevalence rates ranging from 60 to 90% in children and 26 to 85% in adults. Currently, there is a dearth of multiepitope vaccines (MEVs) specifically designed to combat *S. mutans*. To address this gap, we employed an immunoinformatics approach for MEV design, identifying five promising vaccine candidates (PBP2X, PBP2b, MurG, ATP-F, and AGPAT) based on antigenicity and conservation using several tools including CELLO v.2.5, Vaxign, v2.0, ANTIGENpro, and AllerTop v2.0 tools. Subsequent identification of linear B-cell and T-cell epitopes by SVMTrip and NetCTL/NetMHC II tools, respectively, guided the construction of a MEV comprising 10 Cytotoxic T Lymphocyte (CTL) epitopes, 5 Helper T Lymphocyte (HTL) epitopes, and 5 linear B-cell epitopes, interconnected by suitable linkers. The resultant MEV demonstrated high antigenicity, solubility, and structural stability. In silico immune simulations showcased the MEV’s potential to elicit robust humoral and cell-mediated immune responses. Molecular docking studies revealed strong interactions between the MEV construct and Toll-Like Receptors (TLRs) and Major Histocompatibility Complex (MHC) molecules. Remarkably, the MEV–TLR-4 complexes exhibited a low energy score, high binding affinity, and a low dissociation constant. The Molecular Dynamic (MD) simulation analysis suggested that MEV–TLR-4 complexes had the highest stability and minimal conformational changes indicating equilibrium within 40 nanosecond time frames. Comprehensive computational analyses strongly support the potential of the proposed MEV to combat dental caries and associated infections. The study’s computational assays yielded promising results, but further validation through in vitro and in vivo experiments is needed to assess its efficacy and safety.

## 1. Introduction

Dental caries, or tooth decay, is a prevalent global oral health issue affecting over 4 billion people worldwide, imposing significant health and economic burdens [1,2]. This complex condition is primarily driven by *Streptococcus mutans*, a bacterium naturally residing in the oral cavity, particularly in dental plaque. The viridans group streptococci, including *S. mutans*, can comprise up to 80% of early dental plaque. The cariogenicity of *S. mutans* is attributed to three key traits: (i) synthesizing extracellular glucan polymers from sucrose, facilitating biofilm matrix formation, (ii) fermenting carbohydrates into organic acids, and (iii) thriving in acidic environments. These properties promote an exopolysaccharide-rich, low pH milieu conducive to other acidogenic bacteria, aiding in the formation of dental plaque (a multispecies biofilm on tooth surfaces) [3,4]. Beyond the oral cavity, *S. mutans* is associated with several other health disorders. *S. mutans* strains are classified into four capsular rhamnose–glucose polysaccharide serotypes (c, e, f, k), with serotype c being predominant in dental plaque (~75%), followed by serotype e (~20%), and serotypes f and k constituting the remainder (~5%). Serotypes e, f, and k express collagen-binding proteins (Cnm or Cbm), linking them to extra-oral systemic diseases such as sub-acute infective endocarditis (IE), atherosclerosis, cerebral microbleeds, and immunoglobin A (IgA) nephropathy [5]. The etiological significance of *S. mutans* is underscored by the fact that together with staphylococci and enterococci, viridans group streptococci account for up to 90% of all infective endocarditis (IE) cases [6].

Although historically viewed as a childhood disease, dental caries persist into adulthood, with a prevalence of 60–90% in children and 26–85% in adults [7]. The close association between dental caries and *S. mutans* is primarily attributed to the bacteria’s exceptional ability to adhere to tooth surfaces and form biofilms [8]. It metabolizes sucrose to produce acidic byproducts, leading to enamel demineralization and subsequent caries development [9]. The biofilm matrix enables the bacteria to withstand environmental changes in the oral cavity, including pH fluctuations, nutrient availability, and aerobic-to-anaerobic transitions [9,10]. This adaptability extends to enhancing resistance against mechanical host-clearance and certain antimicrobial agents, bolstered by the production of multiple glucan proteins [11]. Biofilms, with their structured matrix, exhibit resilience to hostile conditions and are implicated in various other systemic conditions [12].

Currently, the management of dental caries mainly relies on antimicrobial agents including chlorhexidine, fluoride, quaternary ammonium salts, and iodine, alongside antibiotics to combat various oral pathogens [13]. However, prolonged use of these agents raises concerns, notably contributing to the development of antimicrobial resistance and causing adverse effects on various oral components including mucosal cells, keratinocytes, osteoclasts, osteoblasts, and blood cells [11]. These side effects are considered a significant contemporary health issue [14]. As such, *S. mutans* remains a formidable adversary in oral health, and effectively addressing this problem requires a multi-faceted approach involving innovative research, preventive measures, and therapeutic strategies. In the last decade, several experiments have been conducted to develop vaccines for preventing and treating dental caries with immunotherapy [15,16]. Caries vaccination has emerged as a highly promising method for preventing dental caries, presenting the advantage of offering long-term defense [17]. *S. mutans* employs various surface proteins such as glucan-binding proteins (Gbps), glycosyltransferases (Gtfs), collagen-binding proteins, protein antigen c, and amyloids to facilitate biofilm formation and maturation [18,19]. Surface proteins are believed to be crucial in bacteria–host interactions, leading to a focus on these proteins in most caries vaccine designs. A caries vaccine, like other vaccines, should be administered before exposure to the infectious agent. Immunization with *S. mutans* antigens is expected to trigger antibody production in saliva, leading to cellular aggregation, which reduces bacterial adherence to tooth surfaces [20].

Studies on dental caries vaccines in animal models have shown promise in an active immunization approach. These studies have demonstrated that immunization via subcutaneous, oral, intranasal, and topical routes induces salivary IgA and serum IgG responses, which play key roles in immunity. For example, subcutaneous immunization with cell surface adhesion proteins (Antigen I/II) in Rhesus monkeys led to a 70% reduction in caries [21]. Studies have shown that oral delivery of anti-*S. mutans* vaccines using liposomes can stimulate gut-associated lymphoid tissue (GALT) and enhance mucosal immune responses leading to reduced caries and bacterial colonization in rats [22]. Few studies have assessed the effectiveness of active immunity against *S. mutans* in humans, focusing primarily on oral administration. The ingestion of capsules containing *Streptococcus sobrinus* effectively induced salivary sIgA against *S. mutans* [23]. However, a follow-up study found no salivary IgA response when administering heat-killed *S. sobrinus* cells orally [24]. Oral immunization with *S. mutans* glucosyltransferase (GTF) increased salivary IgA in seven subjects, with similar results observed when the antigen was administered intranasally or topically to the tonsils [15,25].

Dental caries vaccines have shown effectiveness, but cross-reactivity with heart and skeletal muscle tissues is a major drawback, likely due to whole-cell vaccines. Traditional anti-caries vaccine development faces various challenges, such as the requirement for identifying antigens with dual characteristics of immunogenicity and specificity to *S. mutans*. There is the additional complication of inducing a strong and long-lasting immune response against the bacteria in the particular oral environment [26]. Given these challenges, it is critical to explore alternative immunotherapies against dental caries. A study attempted to reduce this issue with subunit vaccines, but they were less immunogenic [27]. A recent study has reported dextransucrase antibodies to reduce acid production and bacterial hydrophobicity, exhibiting anti-cariogenic activity without harmful cross-reactivity [28].

The evolution of reverse vaccinology and immunoinformatics has brought attention to the multiepitope vaccine (MEV) since such vaccines have higher immunogenicity, low allergenicity, cost-effectiveness, time-efficiency, and a lower risk of side effects [29,30]. Furthermore, the MEV holds the advantage of concurrently eliciting diverse immune responses, encompassing humoral, innate, and cellular responses, surpassing the capabilities of monovalent vaccines [31]. MEVs developed using immunoinformatic have demonstrated in vivo prophylactic properties, with many progressing to the clinical trial stage [32]. Presently, the MEV approach has shown promise against a spectrum of pathogens, including *Mycobacterium tuberculosis* [33], *Serratia marcescens* [34], group B *Streptococcus* [35], *Streptococcus suis* [29], *Staphylococcus aureus* [36], Monkeypox virus [37], and SARS-CoV-2 [38]. While various studies have explored MEVs for numerous bacterial pathogens, limited literature exists for MEVs targeting *S. mutans*. Therefore, this study aims to develop a universal MEV candidate against *S. mutans* using immunoinformatic approaches. Five highly conserved membrane proteins in *S. mutans* were identified, and their antigenicity and B and T cell epitopes were analyzed. The top-ranking epitopes were assembled to construct the MEV, which underwent further analysis for binding affinity towards human Toll-like receptors (TLRs), MHC molecules, and immune stimulation through bioinformatics methods. The stability of the MEV was further validated through molecular dynamics simulations, revealing that the designed MEV established stable interactions with human immune receptors, thereby enabling the stimulation of a robust host immune response.

## 2. Materials and Methods

**Figure 1:**
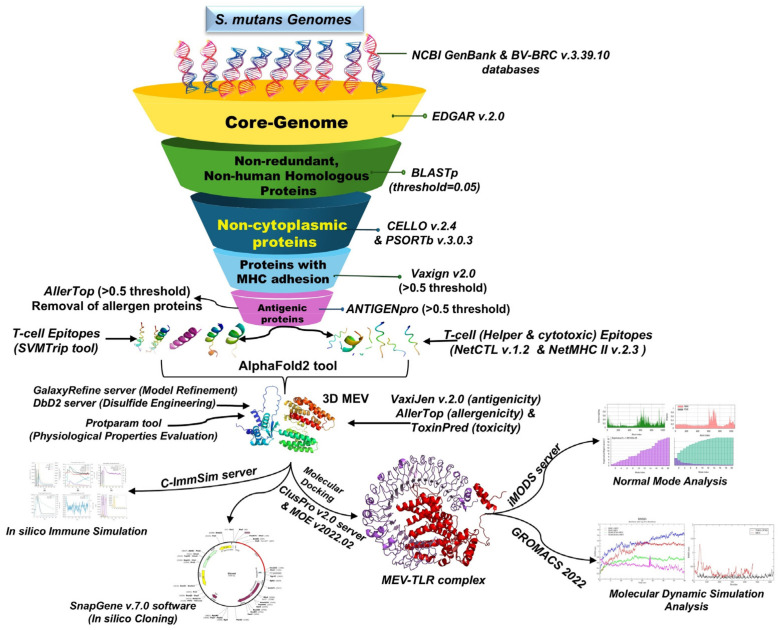
A schematic overview of the immunoinformatics approach implemented in this study for constructing a novel MEV designed to target *S. mutans*.

The process used to create a novel MEV that targets *S. mutans* is shown in Figure 1 and is further explained in the section that follows.

### 2.1. Retrieval of Streptococcus mutans Protein Sequences

In this study, 18 whole-genome sequences of *S. mutans* strains associated with human infections were retrieved from the NCBI-Genbank database (http://www.ncbi.nlm.nih.gov/genbank/; accessed on 7 February 2023) [39] and Bacterial and Viral Bioinformatics Resource Center (BV-BRC) v.3.39.10 (https://www.bv-brc.org/; accessed on 7 February 2023) (Table S1). The core genome was generated using the EDGAR v.2.0 software framework (https://edgar3.computational.bio.uni-giessen.de/; accessed on 20 February 2023) [40]. Paralog sequences within the core proteome were refined through the CD-HIT suite, employing a cutoff value of 0.7 (70%) for clustering similarity [41]. To eliminate analogous protein sequences from *Homo sapiens* within the core proteome, BLASTp [42] was employed with default parameters. Subsequently, non-homologous protein sequences, devoid of any matches, were selected for further analysis.

The protein sequences were used for the identification of subcellular location using CELLO v.2.5 (http://cello.life.nctu.edu.tw; accessed on 29 February 2023) [43] and PSORTb v.3.0.3 (https://www.psort.org/psortb; accessed on 29 February 2023) [44] servers. The proteins localized in the non-cytoplasmic regions were selected to predict the MHC (class I and II) binding affinity and the number of transmembrane helices using the Vaxign v2.0 tool (https://violinet.org/vaxign2; accessed on 12 March 2023) [45]. The MHC adhesion probability of >0.5 value was selected for identification of antigenicity and allergenicity using ANTIGENpro (http://scratch.proteomics.ics.uci.edu; accessed on 24 March 2023) [46] and AllerTOP v.2.0 server (http://www.ddg-pharmfac.net/AllerTOP; accessed on 26 March 2023) [47], respectively. A threshold of >0.5 provides a balance between sensitivity (true positives) and specificity (true negatives) in predicting antigenicity and allergenicity. This value increases the probability of accurate classification, indicating that a protein is more expected to be antigenic or allergenic.

### 2.2. Prediction of B-Cell Lymphocyte Epitopes

The identified vaccine candidate proteins were analyzed for 20-mer linear B-cell lymphocyte epitopes using SVMTrip (http://sysbio.unl.edu/SVMTriP/prediction.php; accessed on 4 April 2023) [48]. The linear 20-mer B-cell lymphocyte epitopes with a score greater than 0.8 were selected due to their high probability of antigenicity, a score greater than 0.8 indicating strong prediction, reducing false positives. The SVMTrip utilized the latest Immune Epitope Database and Analysis Resource (IEDB) to enhance the prediction of B-cell linear epitope locations in the provided protein sequence, employing a Support Vector Machine (SVM) model [33,49]. The ToxinPred server (https://webs.iiitd.edu.in/raghava/toxinpred/design.php; accessed on 9 April 2023) [50] was employed to assess the toxicity of B-cell epitopes. The antigenicity of B-cell epitopes was determined through the VaxiJen v.2.0 server at a threshold value of 0.4 [45]. Allergenicity analysis of B-cell epitopes was conducted using the AllerTOP v.2.0 server [47], which transforms protein sequences into uniform, equal-length vectors through auto cross-covariance. Additionally, the Epitope Conservancy Analysis tool was employed to ascertain the conservancy degree of predicted epitopes, utilizing the Immune Epitope Database (IEDB) [51].

### 2.3. Prediction of Cytotoxic T Lymphocyte Epitopes

The pivotal step in subunit vaccine design for attaining immunologic significance in multi-epitope vaccine candidates involves the identification of potential epitopes. Employing the NetCTL v.1.2 server (https://services.healthtech.dtu.dk/services/NetCTL-1.2/; accessed on 14 April 2023) [52], we analyzed cytotoxic T lymphocyte epitopes within vaccine candidate protein sequences. Employing a cut-off value of 0.75, this study selected 12 MHC class I supertypes to predict 9-mer CTL epitopes [53]. The cut-off value ≥ 0.75 maximizes accuracy and prioritizes stronger candidates. Peptides above this value are more estimated to be recognized by CTLs, making them crucial for designing vaccines or immunotherapies and minimizing false positives. The NetCTL v.1.2 server integrates the prediction of peptide MHC class I binding, proteasomal C-terminal cleavage, and transporter associated with antigen processing (TAP) transport efficiency [54].

### 2.4. Prediction of Helper T Lymphocyte Epitopes

The NetMHC II v.2.3 server (https://services.healthtech.dtu.dk/services/NetMHCII-2.3/; accessed on 18 April 2023) [55] was applied to identify the binding of 15-mer peptides to a 7-allele HLA (human leukocyte antigen) such as HLA-DRB1*01:01, HLA-DRB1*01:03, HLA-DRB1*03:01, HLA-DRB1*04:01, HLA-DRB1*04:02, HLA-DRB1*04:04, and HLA-DRB1*07:01 using artificial neural networks (ANNs). The threshold values for stronger and weaker binding were established at 2% and 10%, respectively [54].

The identified CTL and HTL epitopes were thoroughly evaluated for antigenicity, allergenicity, toxicity, and conservancy using Vaxijen 2.0, AllerTOP 2.0, ToxinPred, and Epitope Conservancy Analysis, respectively.

### 2.5. Prediction of Interferon-Gamma (INF-ꝩ), Tumor Necrosis Factor-Alpha (TNF-α), and Interleukin-10 (IL-10)

The chosen HTL epitopes were employed to predict the ability of HTL epitopes to elicit INF-ꝩ, TNF-α, and IL-10 immune responses through the utilization of IFN-Pred (http://crdd.osdd.net/raghava/ifnepitope/predict.php; accessed on 21 April 2023) [56], TNFepitope Server (https://webs.iiitd.edu.in/raghava/tnfepitope/ predict_human.php; accessed on 21 April 2023) [57], and IL-10Pred (https://webs.iiitd.edu.in/raghava/il10pred/predict3.php; accessed on 21 April 2023) [58], respectively, employing the Support Vector Machine (SVM)-based method [59].

### 2.6. Construction of MEV Candidate Sequence

The MEV for *S. mutans* was assembled using highly immunogenic, antigenic, non-toxic, and non-allergenic epitopes as structural components. CTL, HTL, and linear B-cell epitopes were assembled to form the MEV structure, incorporating linkers AAY (Ala-Ala-Tyr), GPGPG (Gly-Pro-Gly-Pro-Gly), and KK (bi-lysine) for interconnection [60]. Additionally, the 50S ribosomal L7/L12 protein, specifically Locus RL7_MYCTU (accession number: P9WHE3), was incorporated as an adjuvant at the N-terminal of the vaccine construct, linked via an EAAAK linker, aiming to augment the immunogenicity of the multiepitope vaccination [49].

### 2.7. Evaluation of Physiological, and Solubility Properties

The physiological properties of the MEV construct were evaluated by using Protparam (https://web.expasy.org/protparam/; accessed on 9 May 2023) [61]. This tool computed parameters such as the molecular weight, amino acid composition, atomic composition, theoretical pI, extinction coefficient, instability index (II), estimated half-life, aliphatic index, and grand average of hydropathicity (GRAVY) [62]. Furthermore, the solubility of the vaccine design was examined using the SOLpro website [46]. The SVM architectural method is employed by this tool to predict the protein’s probability of achieving solubility upon overexpression in *Escherichia coli* [63].

### 2.8. Prediction of Secondary Structure and Tertiary Structure

To generate the secondary structure of the MEV construct, PBDsum (https://www.ebi.ac.uk/thornton-srv/databases/pdbsum/Generate.html; accessed on 12 May 2023) [64] was employed. This tool utilizes a highly precise prediction algorithm and two feed-forward neural networks to analyze PSI-BLAST (Position-specific integrated-BLAST) output [65].

For the ab initio 3D structure of the MEV construct, AlphaFold v.2 integrated into the Galaxy Europe server (https://usegalaxy.eu; accessed on 12 May 2023) [66] was utilized, employing the monomer preset model from AlphaFold’s comprehensive database. The resulting 3D structures underwent evaluation using ERRAT, PROCHECK, and Ramachandran plot details were assessed through SAVES v.6.1 tool (https://saves.mbi.ucla.edu/; accessed on 18 May 2023) [34].

### 2.9. Refinement and Validation of Tertiary Structure

The refinement of the 3D model for the MEV construct generated by AlphaFold v.2 was conducted using the GalaxyRefine server (https://galaxy.seoklab.org/cgi-bin/submit.cgi?type=REFINE; accessed on 19 May 2023). Employing a successful refinement technique validated in CASP10-based experiments [67], the GalaxyRefine server is targeted to enhance the local structure quality.

Ensuring the validation of the tertiary structure is essential in vaccine development, as it helps identify potential issues with the predicted model [68]. The ProSA-web server (https://prosa.services.came.sbg.ac.at/prosa.php; accessed on 22 May 2023) was used to validate the refined 3D model of the MEV construct. This involved a comparison between the initial 3D structure and the refined structure of the vaccine candidate. The overall quality of the improved MEV candidate structure was evaluated by employing the ProSA-web server, expressed as a Z-score [69]. If the Z-score falls outside the typical range for native proteins, it suggests potential structural flaws. The non-bonded atom–atom interactions associated with the revised 3D model of the vaccine construct were analyzed using the ERRAT web server [70]. Further validation of the refined 3D model for the MEV candidate was conducted by generating a Ramachandran plot using the PROCHECK tool (https://saves.mbi.ucla.edu/; accessed on 22 May 2023). Ramachandran plot statistics provide insights into the distribution of amino acid residues in favorable, allowed, and disallowed regions. A score exceeding 85% is considered indicative of an acceptable 3D model [34].

### 2.10. Prediction of Discontinuous B-Cell Epitopes

In the refined 3D model of the MEV construct, Ellipro (http://tools.iedb.org/ellipro/; accessed on 26 May 2023) was employed to identify discontinuous or conformational B-cell epitopes. ElliPro assigns a protrusion index (PI) value to each projected epitope, offering insights into the ellipsoid shape of the protein and the isoelectric point (pI) of the residues, including nearby cluster residues. Default parameters were used for epitope prediction, with a minimum score of 0.5 and a maximum score of 0.6 [71].

### 2.11. Disulfide Engineering

Disulfide engineering increases the stability of the protein structure in the vaccine build by including new disulfide bonds through cysteine modification of protein structure residues [72]. To introduce the disulfide linkages in the refined MEV structure, the Disulfide by Design2 (DbD2) server (http://cptweb.cpt.wayne.edu/DbD2/index.php; accessed on 30 May 2023) was employed to propose pairs of residues suitable for mutation [73].

### 2.12. In Silico Immune Simulation

The *in silico* immune response profiling of the MEV construct was executed using the C-ImmSim server (http://150.146.2.1/C-IMMSIM/index.php; accessed on 2 June 2023). This server delves into the humoral and cellular profile of the mammalian immune system in response to the MEV construct [74]. Following literature recommendations [54], three vaccination doses were administered at intervals of 28 days. The simulation involved a total of 1000 steps, with injection time steps set at 1, 84, and 168. Default settings were applied for the remainder of the simulation.

### 2.13. Molecular Docking Simulation

In this study, we employed the ClusPro v.2.0 server (https://cluspro.org/help.php; accessed on 12 July 2023) and MOE v.2022.02 (Molecular Operational Environment) software to evaluate the binding affinity of a meticulously refined 3D model of an MEV construct and its interaction with immune receptors. The ClusPro v.2.0 server is a versatile and user-friendly tool designed for the prediction of protein–protein interactions and the elucidation of structural intricacies, featuring an intuitive user interface. It employs Fast Fourier Transform correlation to assess multiple docked conformations, using basic scoring functions within a comprehensive multistage protocol. This protocol includes flexible and rigid docking, energy-based filtering, clustering-based ranking, and energy minimization refinement [75].

To investigate the interactions, protein–protein docking was performed between the refined 3D model of the MEV construct and TLRs, specifically TLR-2 (PDB ID: 6NIG), TLR-3 (PDB ID: 1ZIW), TLR-4 (PDB ID: 4G8A, 3FXI). Additionally, the refined 3D model of the MEV construct was docked with MHCs, such as MHC-I (MHC-I, HLA-A*02:01; PDB: 1AKJ) and MHC-II (MHC-II, HLA-DR1; PDB: 1KG0). Before initiating docking, the PDB structures of TLR-2, TLR-3, TLR-4, MHC-I, and MHC-II were pre-processed to remove connected ligands, heteroatoms, and all chains other than A [76].

The docked complexes generated by the ClusPro v.2.0 server were further analyzed using the PRODIGY web server (https://rascar.science.uu.nl/prodigy/; accessed on 26 July 2023). PRODIGY was employed to estimate the binding affinity (kcal/mol) and the dissociation constant at 37 °C [77]. The output of PRODIGY is presented in the form of Gibbs free energy (∆G), a pivotal thermodynamic parameter that gauges the energetic feasibility of the binding affinity of a biological complex. Lower ∆G values are indicative of a higher likelihood of the biomolecules forming a biologically meaningful complex [78]. Furthermore, protein–protein docking was conducted using MOE v.2022.02 software to predict the optimal pose of the MEV constructs for efficient binding at the receptor binding domain. MOE v.2022.02 software employed a rigid body refinement approach to enhance the accuracy of the predictions made by the ClusPro v.2.0 server. The vaccine–receptor complexes exhibiting the lowest Gibbs free energy (ΔG) in kcal/mol were selected for further analysis, as these complexes exhibit the most thermodynamically stable, favorable interaction, and spontaneous binding process [79]. The complexes were visualized using the PBDSum server [80].

### 2.14. Molecular Dynamics Simulation

The MD simulation analysis of the MEV construct, which yielded significant results in the molecular docking study, was conducted using GROMACS 2022 [81] to assess the stability and flexibility of the docked MEV–receptor complex. The topology of the complex was generated using the ff99SB force field [82], and the complex was placed in a cubic box with a minimum 3 nm distance from the edges, solvated with the TIP3P water model, and neutralized the surface charge of the molecular structure with sodium and chloride ions. The steepest descent algorithm was used to minimize energy. This was followed by a 100 ps NVT equilibration at 300 K using the Berendsen thermostat, and a 100 ps NPT equilibration at 300 K and 1 bar with the Parrinello–Rahman barostat, employing the LINCS algorithm to constrain hydrogen bonds. A 40 ns MD simulation was then executed, with stability assessments performed using RMSD (root square mean deviation) and RMSF (root mean square fluctuation) analyses through the *gmx rms* and *gmx rmsf* modules, respectively, and visualized with the *xmgrace* module.

Additionally, iMODS web server (https://imods.iqf.csic.es/; accessed on 3 December 2023) was used to analyze the deformability and rigidity in the residue of MEV–TLR complexes by the normal mode analysis (NMA) [83]. iMODS calculates stability for both native and mutant structures using NMA, assessing parameters such as the main-chain elastic network model, covariance matrix, variance, eigenvalue, B-factor, and deformability [34].

### 2.15. Codon Optimization and In Silico Cloning

The Backtranseq server (https://www.ebi.ac.uk/jdispatcher/st/emboss_backtranseq/; accessed on 7 December 2023) was used to reverse-transcribe the final sequence of the MEV construct into a nucleotide sequence. The Java Codon Adaptation Tool (Jcat) server (https://bio.tools/jcat; accessed on 7 December 2023) was utilized for reverse translation, codon optimization, and the determination of the codon adaptation index (CAI) value and GC content of the MEV construct in *Escherichia coli* K12 [84]. Three features were integrated to preclude the presence of rho-independent transcription terminators, prokaryotic ribosome binding sites, and restriction enzyme cleavage sites. Additionally, the DNA sequence encoding the MEV construct had *XbaI* and *XhoI* restriction sites added to its 3′ and 5′ ends, respectively. Ultimately, the optimized sequence of the final MEV construct, complete with restriction sites, was inserted into the pET28a (+) expression vector between the *XhoI* and *XbaI* restriction sites using SnapGene v.7.0 software [49].

## 3. Results

### 3.1. Retrieval of Streptococcus mutans Protein Sequences

The genomes of eighteen *S. mutans* generated 2761 CDS as the core-proteome, of which five proteins were identified as the best vaccine candidate proteins that satisfied all the vaccine characterization criteria (Table S2).

### 3.2. Prediction of B-Cell Lymphocyte Epitopes

The SVMTrip server identified 14 recommended 20-mer linear B-cell epitopes with > 0.8 scores from the five vaccine candidate proteins including PBP2X (penicillin-binding protein 2X), PBP2b (penicillin-binding protein 2b), MurG (UDP-N-acetylglucosamine--N-acetylmuramyl-(pentapeptide) pyrophosphoryl-undecaprenol N-acetylglucosamine transferase), ATP-F (ATP synthase b), and AGPAT (1-acylglycerol-3-phosphate O-acyltransferase). The B-cell epitopes with high binding affinity scores of 1, antigenic, non-toxigenic, and non-allergenic properties, and conservancy were chosen for the vaccine design (Table 1).

### 3.3. Prediction of Cytotoxic T Lymphocyte Epitopes

The NetCTL 1.2 server identified 77 (9-mer) CTL epitopes within 5 vaccine candidate proteins, adhering to cut-off values of 0.75. From these predicted CTL epitopes, a selection of 10 epitopes was made based on their notable scores in MHC class I binding affinity, antigenicity, lack of allergenicity, and non-toxicity (Table 2).

### 3.4. Prediction of Helper T Lymphocytes Epitopes

The NetMHC II server identified a total of 147 numbers of 15-mer HTL epitopes from 5 vaccine candidate proteins that had a binding affinity to HLA-DRB1*01:01, HLA-DRB1*01:03, HLA-DRB1*03:01, HLA-DRB1*04:01, HLA-DRB1*04:02, HLA-DRB1*04:04, and HLA-DRB1*07:01 (Supplementary File S1).

### 3.5. Prediction of Interferon-Gamma (INF-ꝩ), Tumor Necrosis Factor-Alpha (TNF-α), and Interleukin-10 (IL-10)

Among the forecasted HTL epitopes, a selection was made of five 15-mer HTL epitopes for each vaccine candidate protein, chosen for their high binding affinity, antigenicity, non-allergenicity, non-toxicity, and their capacity to induce INF-ꝩ. Specifically, three HTL epitopes originating from vaccine candidate proteins (PBP2b, ATP-F, and AGPAT) displayed the capacity to induce TNF-α, while vaccine candidate proteins (PBP2b and AGPAT) demonstrated the ability to induce IL-10 (Table 3).

### 3.6. Construction of MEV Candidate Sequence

**Figure 2:**
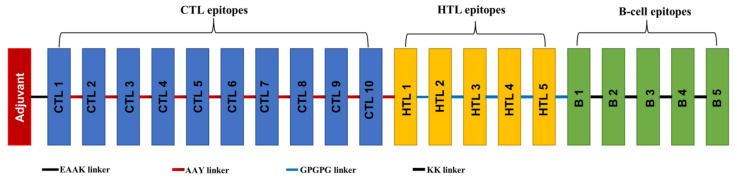
A graphical representation of the final MEV construct. The 464-amino acid peptide sequence, with the adjuvant at its N-terminal (red), is linked to the multiepitope sequence through an EAAAK linker (shaded in gray). CTL epitopes are connected by AAY linkers (red), HTL epitopes with GPGPG linkers (blue), and B-cell epitopes with KK linkers (black).

The MEV candidate was composed of ten CTL epitopes, five HTL epitopes, and five linear B-cell epitopes seamlessly linked by AAY, GPGPG, and KK linkers, respectively. Additionally, the N-terminal of the vaccine construct sequence incorporated the 50S ribosomal L7/L12 protein of *M. tuberculosis* (adjuvant) through the EAAAK linker (Figure 2).

### 3.7. Evaluation of Physiological, and Solubility Properties

The final MEV construct consisted of 464 amino acids, exhibiting a molecular weight of 49.7 kDa. Theoretical estimations attributed an isoelectric point (pI) of 8.20 and a GRAVY value of −0.030 to the vaccine construct. With an instability index (II) value of 21.81, the MEV construct exhibited stability. Notably, the construct revealed a high aliphatic index score of 87.67, indicative of considerable thermostability. The half-life of the MEV construct was computationally predicted using the ProtParam tool, showing 30 h in mammalian reticulocytes, over 20 h in yeast, and 10 h in *E. coli*, signifying robust stability across different environments. Furthermore, the SOLpro server predicted a high solubility probability of 0.898173 for the MEV construct. Evaluation from the AllerTOP v.20 and ANTIGENpro servers categorized the MEV construct as non-allergenic and antigenic (0.556701), respectively. According to the VaxiJen v.2.0 tool, the MEV construct attained a score of 0.5464, suggesting its probable antigenic nature in a bacterial model, utilizing a threshold of 0.4.

### 3.8. Prediction of Secondary Structure and Tertiary Structure

The secondary structure analysis of the MEV construct conducted by PDBsum revealed the presence of 21 helices, 37 helix–helix interactions, 10 beta-turns, and 9 gamma-turns (Figure S1). The tertiary structure of the MEV construct modeled by AlphaFold v.2 tools resulted in five 3D models. Among these 3D models, the Rank 1 model was found to be the most confident model based on the ERRAT and Ramachandran plot (PROCHECK) evaluations. This 3D model had an overall quality factor, ERRAT of 96.5087, 87.5% residues in the favorable regions of the Ramachandran plot, 11.8% in additional allowed regions, 0.5% in generously allowed regions, and 0.2% in the disallowed regions (Figure 2).

### 3.9. Refinement and Validation of Tertiary Structure

**Figure 3:**
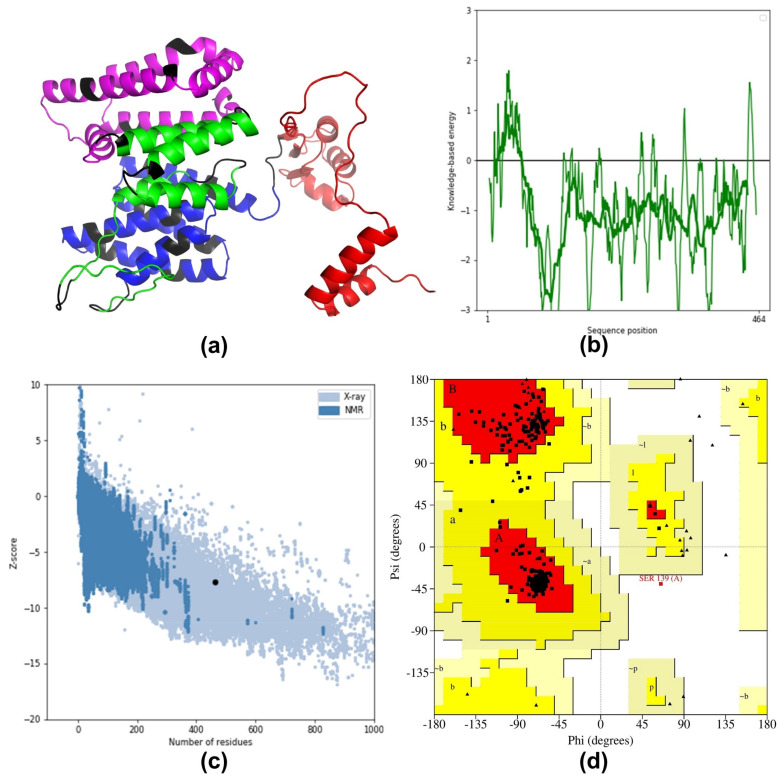
3D structure modeling and validation of MEV. (**a**) Refined 3D structure of MEV generated by AlphaFold v.2, in which adjuvant (50S ribosomal L7/L12 protein of *M. tuberculosis*) is represented by a red color, B−cell epitopes by a magenta color, HTL epitopes by a green color, CTL epitopes by a blue color, and the linkers represented by a black color. (**b**) Energy plot model of the 3D structure of MEV refined by GalaxyRefine. (**c**) Z−score graph via ProSA web showing that the GalaxyRefine modeled 3D structure corresponds to X−Ray crystallographic determined structure for the protein of same sizes. (**d**) The Ramachandran plot showing the distribution of phi (Φ) and psi (Ψ) angles for residues in the refined 3D model of the MEV. Black squares denote non-glycine, non-proline residues, predominantly located in the most favored regions (A, B, L). Glycine residues, known for their flexibility, are represented by triangles, while proline residues, with their restricted conformations, are shown as red circles. The plot reveals that 97.1% of residues fall within the most favored regions, 2.7% in additional allowed regions (a, b, l, p), and one residue (0.2%) is in a disallowed region.

The GalaxyRefine server-generated five top models from the initial Rank 1 model. Following an evaluation of structural quality across all the 3D models, the Model 4 exhibited the most significant scores in terms of GDT-HA (0.9623), RMSD (0.378), MolProbity (1.190), Clash score (4.1), Poor rotamers (0.3), and Rama favored (98.5) (Table S3). This model was selected for further validation, employing the z-scores from ProSA, ERRAT, and Ramachandran plots via PROCHECK. The ProSA server calculated a Z-score of −7.66 for the initial model and −7.72 for the refined model of the MEV construct (Figure 3). Refined Model 4 exhibited an enhanced overall quality factor of 96.5432. The Ramachandran plot analysis conducted via PROCHECK revealed 97.1% of residues situated in the most favored regions, 2.7% in other allowed regions, no residues in generously allowed regions, and only 0.2% in the disallowed regions (Figure 3).

### 3.10. Prediction of Discontinuous B-Cell Epitopes

The refined tertiary structure of the MEV construct underwent analysis to identify discontinuous or conformational B-cell epitopes. Using the ElliPro server, a total of 227 residues were identified, forming 11 discontinuous B-cell epitopes with scores ranging from 0.509 to 0.846. The confirmation sizes of these B-cell epitopes vary from 5 to 54 residues. Notably, Table S4 and Figure S3 depict the B-cell discontinuous epitopes with a score value surpassing 0.70.

### 3.11. Disulfide Engineering

The refined model of the MEV construct was engineered by the addition of new disulfide bonds using the DbD2 server. This sever predicted that the 39 residue pairs had the potential to form disulfide bonds, in which 4 residue pairs were selected for disulfide bond formation including ALA250–ALA262, ALA325–SER345, GLY335–VAL338, and Leu435–ALA439. The residue pairs were analyzed based on the x3 angle between −87° and +97°. The wild-type and mutant MEV construct models are shown in Figure S4.

### 3.12. In Silico Immune Simulation Response

**Figure 4:**
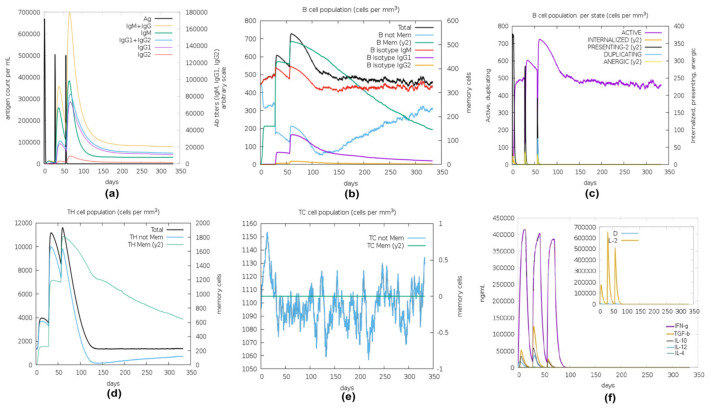
The spectra of immune responses in response to the three doses of MEV injections. (**a**) The immune response of antibodies to the designed MEV. (**b**) Dynamics of B-cell populations. (**c**) Distribution of B-cell populations post-injection. (**d**) Changes in T-helper cell populations after three doses. (**e**) Evolution of T-cytotoxic cell populations. (**f**) The principal spectrum plot illustrates cytokine levels post-injections. The inset plot showcases IL-2 levels alongside the Simpson index; an increase in the D level anticipates the emergence of distinct epitope-specific dominant T-cell clones.

The C-ImmSim server was used to analyze the immune simulation response triggered by the MEV. After each administration of the vaccine dose, there was a significant rise in the antigen count level—6.8 × 10^5^ per mL on the 1st dose and ~5 × 10^5^ per mL on 2nd and 3rd doses that neutralized on the 5th day of injection. This was followed by a rise in the production of secondary immunological (IgM + IgG) response to ~1.8 × 10^5^ titer scale per mL after ~5 days of the 3rd dose injection (Figure 4a). The level of IgM elevated to 1.0 × 10^5^ on an arbitrary scale after ~5 days of the 3rd dose injection. Also, the population of IgG1+IgG2 reached a peak value of ~8 × 10^4^ on an arbitrary scale after ~5 days of the 3rd dose injection (Figure 4a). Elevation of antibody secretion after secondary and tertiary immunological responses, resulting in the production of B-cell population, intensified antigen clearance upon successive exposures (Figure 4b). The total B-cell population rose to 600 cells/mm^3^ after 25 days of the 1st dose and further amplified, reaching a peak of 720 cells/mm^3^ after 5 days of the 3rd dose and declining gradually after that (Figure 4b). The level of memory B-cells elevated from 150 cells/mm^3^ after the 1st dose and attained the highest peak of 500 cells/mm^3^ after the 3rd dose. The level of memory B-cell up-regulation lasted ~350 days (i.e., the entire simulation period) (Figure 4c). Moreover, a heightened activation of cell-mediated immune response events was observed, driven by an upsurge in the population of active Helper T Lymphocytes (HTL) and Cytotoxic T Lymphocytes (CTL) (Figure 4d,e). The total HTL population increased to 400 cells/mm^3^ after the initial 10 days following the first dose, peaking at 1.16 × 10^4^ cells/mm^3^ after 2 days following the second dose. Furthermore, HTL increased proliferation up to 1.9 × 10^4^ cell/mm^3^ after 12 days of the 3rd dose (Figure 4d). The memory HTL had the highest population of 1.8 × 10^3^/mm^3^ after 2 days of the 3rd dose and maintained its upregulation to 350 days (Figure 4d). The number of the resting CTL population dropped vigorously after the first vaccine dose (Figure 4e). In addition, the level of pro-inflammatory IFN-γ was elevated to 4.2 × 10^5^ ng/mL after 12 days following the 1st dose and slightly decreased to 3.7 × 10^5^ ng/mL after 15 days following the 3rd dose (Figure 4f). However, minimal levels of the anti-inflammatory cytokines IL-10, and IL-2 were apparent (Figure 4f).

### 3.13. Molecular Docking Simulation

The protein–protein docking was performed using the ClusPro server with TLRs as receptors (targets) and refined MEV construct as a ligand to determine the strength of binding energy or affinity of the MEV (antigen) molecule toward the immune receptor molecules. These antigen molecule–immune receptor molecule interactions are essential for transporting antigen molecules and promoting both humoral and cell-mediated immune response pathways.

**Figure 5:**
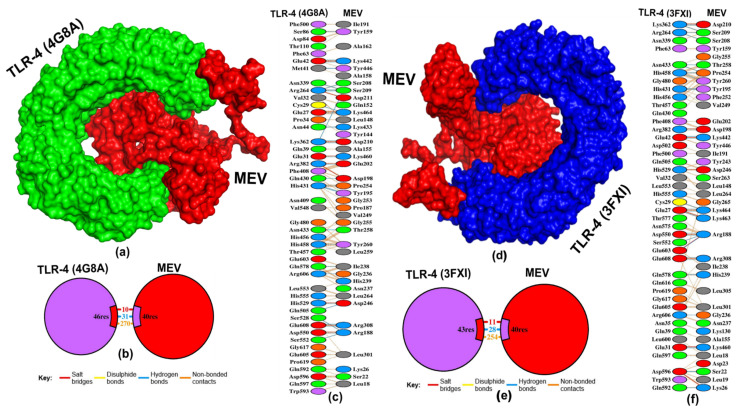
Molecular docking simulation of TLR-4 with MEV. (**a**) The protein–protein docking complex of TLR-4 (4G8A) with MEV. (**b**) The number of bonding and non-bonding contacts in TLR-4 (4G8A)–MEV complex. (**c**) Interacting residues of TLR-4 (4G8A) with the MEV, the blue line denotes H-bond formation. (**d**) The protein–protein docking complex of TLR-4 (3FXI) with the MEV. (**e**) The number of bonding and non-bonding contacts in the TLR-4 (3FXI)–MEV complex. (**f**) Interacting residues of TLR-4 (3FXI) with the MEV, the blue line denotes H-bond formation.

The ClusPro server and MOE software predicted that the designed MEV construct showed low energy scores and strong binding affinities, respectively, toward the TLR-4 (Table 4 and Figure 5). The MEV construct docked with TLR-4 (PDB: 4G8A) and TLR-4 (PDB: 3FXI) by the ClusPro server, suggesting that the MEV–TLR-4 (PDB: 4G8A) complex has low energy score of −1397.7 kcal/mol and displayed the lower binding affinity of −20.3 ΔG (kcal mol-1), and dissociation constant of 1.20 × 10^−15^ kd (M) at 37 °C than the MEV–TLR-4 (PDB: 3FXI) complex (Table 4). The MEV–TLR-4 (PDB: 4G8A) complex formed 31 H-bonds, 10 salt bridges, and 270 non-bonded contacts (Figure 5b). The MEV residues involved in hydrogen (H)-bonds with bond distance < 3.08 Å were LYS464 (2-times), LYS442 (2×), LYS460, TYR446, LYS433, TYR159, SER208, SER209 (4×), ASP210 (2×), GLU202, ASP198, PRO254, TYR195 (2×), THR258, TYR260, ASP246 (2×), ARG188 (3×), ILE238, GLY236, and ARG308 (2×) (Figure 5c). While the MEV formed 28 H-bonds in the MEV–TLR-4 (PDB: 3FXI) complex, with bond distances < 3 Å. The residues of the MEV engaged in H-bond formation comprised LYS 464 (3×), LYS 463, LYS 460, LYS 442 (2×), SER 208, SER209 (2×), ASP210, ASP198, TYR195 (2×), PRO254, THR258, TYR260, PRO254, ARG188 (3×), HIS239, LYS26, SER22, ARG188, GLY236, ARG308 (2×) (Figure 5e,f). Furthermore, the docking of the MEV construct with TLR-4 (PDB: 3FXI) using MOE software predicted a protein–protein interaction with a lower binding affinity of −98.4945 kcal/mol (Table 4).

**Figure 6:**
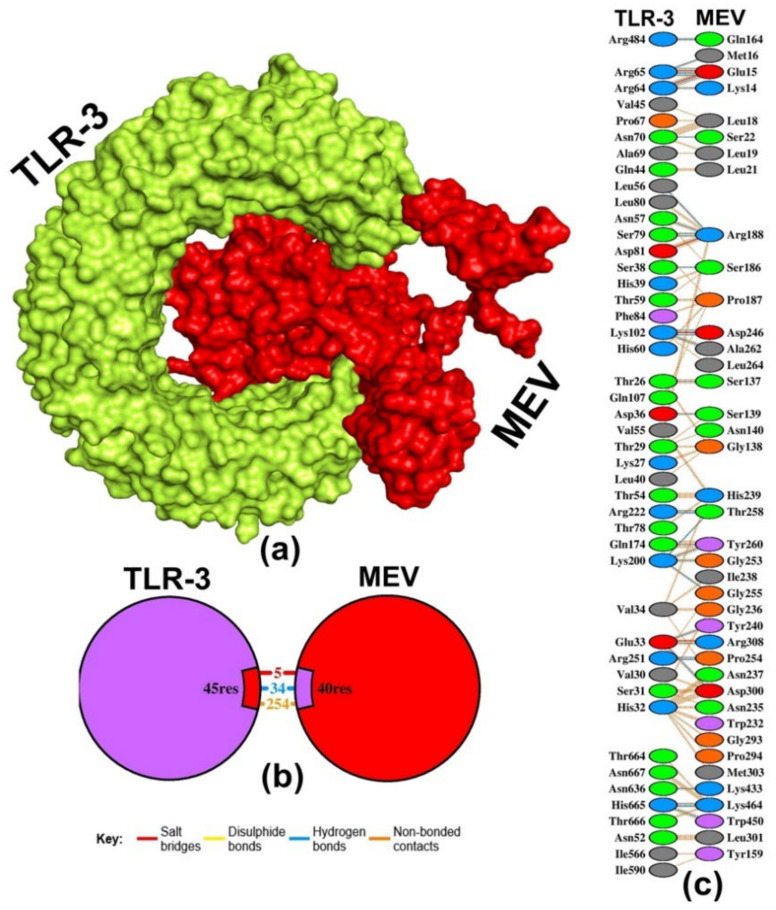
Molecular docking simulations were conducted for TLR-3 with the MEV. (**a**) Depicts the protein–protein docking complex of TLR-3 with MEV. (**b**) Illustrates the number of bonding and non-bonding contacts in the TLR-3–MEV complex. (**c**) The interacting residues of TLR-3 with the MEV, where the blue line signifies hydrogen bond formation.

Moreover, utilizing the ClusPro server, the docking simulation of the MEV construct with TLR-3 revealed exceptionally low energy scores, reaching −1134.3 kcal/mol. The MEV–TLR-3 complex has the binding affinity of −20 ΔG (kcal/mol) and dissociation constant of 2.10 × 10^−15^ kd (M) at 37 °C (Table 4 and Figure 6a). The MEV–TLR-3 complex created 34 H-bonds, 5 salt bridges, and 254 non-bonded contacts (Figure 6b). The amino acid residues that engaged H-bonds with a bond distance < 3.22 Å involved SER137, ASP300, TYR240, ARG308, SER139, SER186, ARG188 (4×), GLU15 (4×), LYS14, MET16, SER22, ASP246 (2×), ALA262, GLY253 (2×), THR258 (3×), TYR260 (2×), PRO254 (2×), GLN164, LYS433, LYS464 (2×), and TRP450 (Figure 6c). Furthermore, the MOE software analysis indicated that the binding affinity of the MEV–TLR-3 complex is −81.1515 kcal/mol. Also, the ClusPro predicted that the docking of TLR-2 with MEV construct generated 30 clusters, of which cluster-0 consisted of the highest members (98) with a low energy score of −1114.7 kcal/mol. The evaluation of its binding affinity using the PRODIGY server revealed −11.6 ΔG (kcal/mol), and a dissociation constant of 3.10 × 10^−9^ kd (M) at 37 °C (Table 4). In addition, the MOE software predicted that the MEV–TLR-2 complex has a −70.3042 kcal/mol binding affinity. The MEV–TLR-2 complex generated 12 H-bonds, 3 salt bridges, and 163 non-bonded contacts (Figure S5). The amino acid residues involved in H-bond formation with a bond distance < 2.84 Å were ARG308 (2×), LYS26, ASN237, TYR311, LYS312, SER263, LEU264 (2×), ARG188 (2×), and VAL268 (Figure S5).

**Figure 7:**
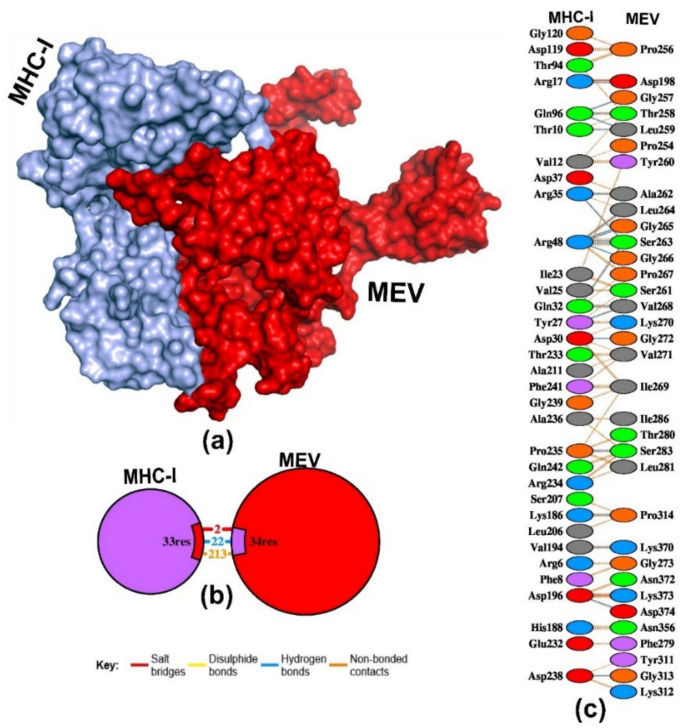
Molecular docking simulation of MHC-I proteins with the MEV. (**a**) The protein-protein docking complex of MHC-I with MEV. (**b**) The number of bonding and non-bonding contact MHC-I–MEV complex. (**c**) Interacting residues of MHC-I with the MEV, the blue line denotes H-bond formation.

Additionally, the MEV–MHC-II complex demonstrated a low ClusPro energy score of −1128.4 kcal/mol compared to the MEV–MHC-I complex (Table 4). However, downstream MOE software and PRODIGY server-mediated assessment of binding affinity revealed the MEV–MHC-I complex to exhibit the lowest binding affinity at −85.4256 kcal/mol, and −15 ΔG, respectively, and possessing low dissociation constant of 2.10 × 10^−12^ kd (M) (Table 4 and Figure 7). The PBDSum showed that the MEV–MHC-I-complex generated 22 H-bonds, 2 salt-bridges, and 213 non-bonded contacts (Figure 7b). The complex involved in H-bonds with bond length < 3.10 Å were LEU259 (2×), ASP198, VAL268 (2×), GLY272, SER263 (4×), ALA262, LEU264, GLY265 (2×), GLY266, THR258, GLY257, PRO314, LYS370, ASP374, SER283, and GLY313 (Figure 7c). Whereas, the MEV–MHC-II complex engaged in six hydrogen bonding, with bond distances less than 3.16 Å, were LYS312 (3×), LEU 264, ARG188, and HIS239 (Figure S6). The MEV–MHC-II complex engaged in six hydrogen bonds (bond distances less than 3.16 Å) with four residues, viz., LYS312 (3×), LEU 264, ARG188, and HIS239 (Figure S6).

### 3.14. Molecular Dynamics Simulation

**Figure 8:**
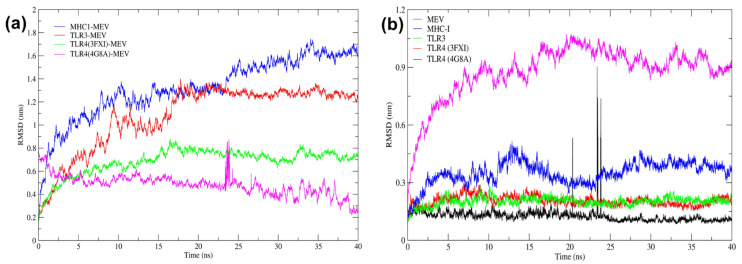
MD-simulation analysis of MEV and receptors for the time duration of 40 ns. (**a**) RMSD plot of MEV docked with TLRs (TLR-3, TLR-4 (4G8A), and TLR-4 (3FXI)) and MHC-I. (**b**) RMSD plot of MEV, TLRs (TLR-3, TLR-4 (4G8A), and TLR-4 (3FXI)) and MHC-I receptors.

The RMSD plot generated from the trajectory data of MD simulations using GROMACS over a 40 ns timescale provided insights into the stability and conformational changes in different protein–vaccine complexes (Figure 8a). The MEV–MHC1 complex (blue) showed an initial increase in RMSD, stabilizing around 1.0 nm. However, minor fluctuations between 1.0 and 1.2 nm towards the end indicated relative structural stability with some conformational changes. The MEV–TLR-3 complex (red) remained relatively stable around 0.8 nm, suggesting a stable interaction with minimal deviations. The MEV–TLR-4 (3FXI) complex (green) gradually stabilized around 0.4 nm with minor fluctuations, indicating a highly stable interaction with minimal conformational changes during the simulation period. The MEV–TLR-4 (4G8A) complex (magenta) also showed high structural stability with RMSD around 0.3 nm and minor small range peaks at 24 ns that quickly returned to the stabilized value. This complex exhibited the lowest RMSD values among all, indicating the highest stability and minimal conformational changes.

Additionally, the RMSD plot was generated from the trajectory data of the MEV construct and unbound receptors to analyze conformational changes and stabilities (Figure 8b). The MEV (magenta line) showed an initial rapid increase in RMSD, stabilizing around 0.9 nm, with minor fluctuations indicating it achieved relative stability. The MHC-I (blue line) stabilized around 0.3 nm, with some fluctuations, suggesting moderate stability. The TLR-3 (green line) stabilized around 0.2 nm, indicating high stability with minimal conformational changes. The TLR-4 (3FXI) (red line) also stabilized around 0.2 nm, showing similar stability to the TLR-3 complex. Finally, the TLR-4 (4G8A) (black line) remained the most stable with RMSD values around 0.1 nm, showing minimal fluctuations and indicating the highest structural stability among the analyzed complexes. Overall, the RMSD analysis demonstrated that while all complexes attained stability, the TLR-4 (4G8A) exhibited the highest stability, followed by TLR-3, TLR-4 (3FXI), MHC-I, and MEV, respectively.

**Figure 9:**
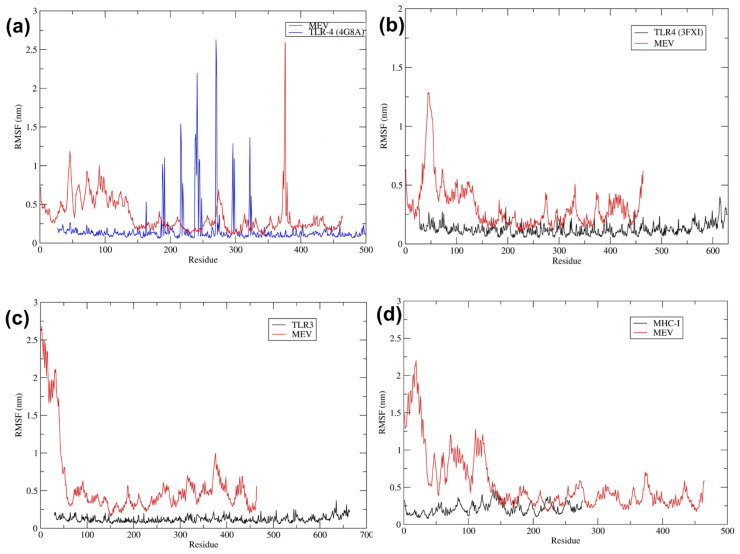
RMSF Analysis of Vaccine and Receptor Complex Stability. (**a**–**d**) The RMSF of MEV–TLR-4 (4G8A), MEV–TLR-4 (3FXI), MEV–TLR-3, and MEV–MHC-I complexes, respectively.

The RMSF analysis of the trajectory data revealed the MEV component consistently exhibits low fluctuations across all complexes, indicating high stability (Figure 9). The RMSF plot for the MEV–TLR-4 (4G8A) complex showed significant fluctuations in several regions, particularly around residues 200–300 and near residue 400, suggesting these regions exhibit higher flexibility when bound to TLR-4 (4G8A). The MEV alone displays lower fluctuations, indicating that binding to TLR-4 (4G8A) induces specific conformational changes and increases flexibility in certain regions (Figure 9a). In the MEV–TLR-4 (3FXI) complex, the RMSF values were relatively low, especially compared to the MEV alone. It suggested a more stable interaction with TLR-4 (3FXI), where the MEV residues exhibit reduced flexibility to stabilize the binding with the MEV structure (Figure 9b). The RMSF plot for the MEV–TLR-3 complex showed higher flexibility in the MEV residues, particularly in the initial 100 residues and around residues 200–300. In contrast, the TLR3 exhibited minimal fluctuations, indicating that while the MEV retains some flexibility, the TLR3 structure remains stable upon binding (Figure 9c). The RMSF analysis of the MEV–MHC-I complex revealed significant flexibility in the MEV residues, especially in the initial 100 residues and around residue 200. The MHC-I receptor itself showed minimal fluctuations, indicating that the binding to MEV does not induce substantial conformational changes in the receptor (Figure 9d).

The stiffness in the mobility and deformability of residues in the MEV–TLR-3 and MEV–TLR-4 (PDB: 4G8A) complexes were analyzed using normal mode analysis (NMA) via the iMODS server, as shown in Figure S7. This study generated conformational morphing trajectories to assess the complex’s compatibility and stability. Structural mobility and flexibility were illustrated through deformability and B-factor plots, identifying non-rigid helical contents by hinge regions (Figure S7a,b) and atomic positional fluctuations (Figure S7c,d). The eigenvalues, indicative of motion stiffness, were 2.339113 × 10^−5^ for the MEV–TLR-3 complex and 1.881333 × 10^−5^ for the MEV–TLR-4 (PDB:4G8A) complex. The lower eigenvalue of MEV–TLR-4 suggested greater stability and flexibility for large-scale conformational changes (Figure S7f). Variance plots showed an inverse relationship with the eigenvalues, with purple and red bars representing individual and cumulative variance (Figure S7g,h). The covariance matrix categorized motions into different modes, with red, blue, and white indicating correlated, anti-correlated, and uncorrelated motions (Figure S7i,j). The elastic network model depicted pairs of atoms connected by springs, with darker gray dots indicating more rigid connections (Figure S7k,l). All these results suggested stable binding interactions with compact conformation and minor fluctuations in the MEV–TLR-3, and MEV–TLR-4 complexes.

### 3.15. Codon Optimization and In Silico Cloning

**Figure 10:**
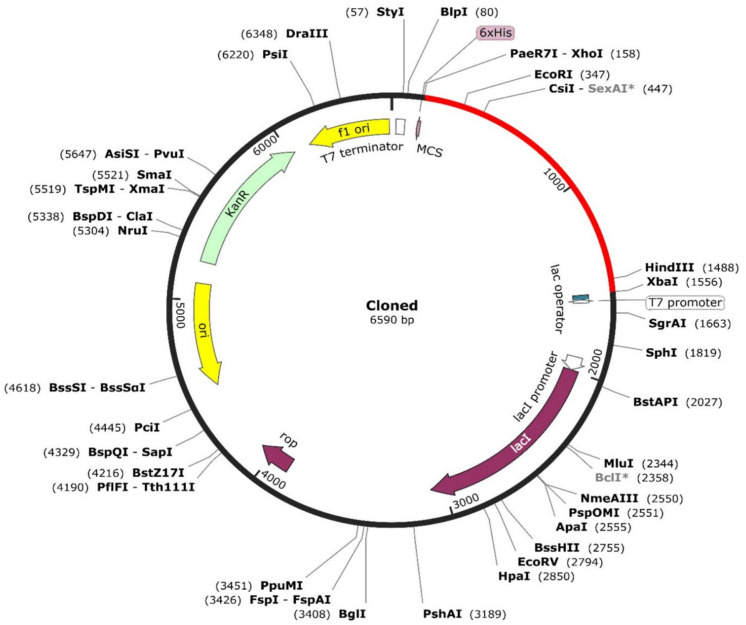
In silico restriction cloning of MEV sequence into the pET28 (+) using SnapGene software. The red color sector represents the coding of MEV gene tags on both ends with *XhoI* and *XbaI* restriction sites. The * symbol on restriction enzymes signifies potential cleavage at non-perfect matches to their recognition sequence.

The Jcat server was employed for codon optimization of the reverse-translated MEV construct against the *E. coli* K12 strain. The optimized sequence achieved a Codon Adaptation Index (CAI) value of 0.99 and a GC content of 49.13%. The refined codon sequence of the MEV construct was strategically tagged with *XhoI* (CTCGAG) at the 3′ terminal and *XbaI* (TCTAGA) at the 5’ terminal to facilitate cohesive ends post-restriction digestion. Subsequently, the recombinant plasmid sequence was meticulously constructed by integrating the adapted codon sequences into the pET28a (+) vector, employing SnapGene v.7.0 software. The resulting circular plasmid, comprising both pET28a (+) and the insert, measured a total length of 6590 base pairs, with the insert contributing 1404 base pairs (Figure 10).

## 4. Discussion

*Streptococcus mutans* is a prominent contributor to dental caries, forming resilient biofilms on tooth enamel that resist antimicrobial agents [85,86,87,88,89]. Biofilm resilience poses risks of severe infections such as bacteremia, atherosclerosis-related plaques and infective endocarditis [90,91,92]. Despite various efforts with antimicrobial agents and monovalent vaccines, significant limitations persist in inhibiting biofilm formation and eliciting long-lasting immune responses [93,94,95]. Advances in immunoinformatics support the development of MEVs that offer cost-effectiveness, improved safety, and the potential to induce diverse immune responses [96,97,98]. These advancements efficiently address the limitations observed in current MEV development approaches against *S. mutans* [99].

The proposed MEV in the present study integrates five highly conserved antigenic proteins (PBP2X, PBP2b, MurG, ATP-F, and AGPAT) identified via a subtractive genomic approach targeting the *S. mutans* core genome. PBP2X and PBP2b, essential in peptidoglycan synthesis, are recognized as vaccine candidates [100,101]. MurG, a bacterial glycosyltransferase, is vital for peptidoglycan biosynthesis, offering potential as an antibiotic target [102]. Previous studies suggested that these proteins exhibit high immunogenic properties and could be used as vaccine candidates in *Neisseria meningitidis* and *Staphylococcus aureus* [103,104]. ATP-F has antigenic potential with non-allergenic, non-toxic, and immunogenic epitopes [105]. AGPAT, involved in lipid synthesis, possesses high antigenic properties [106].

The limited information on these five vaccine candidate proteins did not deter their selection based on high antigenicity as defined by Vaxign [45] and AntigenPro [107]. Important linear B and T-cell epitopes for cellular and humoral immunity were identified by analyzing selected proteins, and these epitopes were subsequently added to the MEV assembly [33]. The predicted HTL epitopes exhibited binding affinities to various HLA alleles, considering geographical variations in HLA expression [108]. HTL epitope selection drives the production of INF-ꝩ, TNF, and IL-10 cytokines in combating pathogens [109]. B-cell epitopes, central to epitope vaccine development, are responsible for the generation and longevity of B-cell mediated immunological memory (memory and plasma cells) [110]. Recognizing these epitopes’ properties, T-cell receptors play a decisive role in immune recognition. As such, the immunotherapeutic and preventive potential of vaccination relies on accurate epitope prediction [111].

In the context of vaccine development, adjuvants play a pivotal role in enhancing and directing the adaptive immune response, improving efficacy, stability, and long-term viability [112,113]. In this study, the *M*. *tuberculosis* 50S ribosomal L7/L12 protein served as a strategically chosen adjuvant, leveraging its documented ability to promote the maturation of dendritic cells, CD4+, CD8+, and INF-producing cells upon stimulation of naïve T-cells, as indicated in previous studies [114]. Using AAY, GPGPG, and KK linkers, respectively, the predicted CTL, HTL, and B-cell epitopes were integrated. AAY provided spatial separation between epitopes, preventing interference, and preserving their structural integrity, while GPGPG maintained epitope conformation for effective immune recognition. Furthermore, the KK linker modulated the vaccine’s charge, which affected how it interacted with immune cells. These linkers, recognized for their flexibility and hydrophilic properties [60] prevent domain disruption [115]. To further enhance the immunogenic properties, EAAAK (a stiff linker) was strategically placed between the adjuvant and the epitope sequences. This modification resulted in an MEV with heightened antigenicity and improved stability scores [116]. The final MEV underwent a comprehensive assessment of its physiochemical properties to facilitate subsequent experimental evaluations of the vaccination and establish an effective setup for both in vitro and in vivo experiments. Designed with a molecular weight of 49.7 kDa, the MEV positioned itself as a promising vaccine candidate, offering ease of cloning, expression in an expression system, and facilitating the purification process [117]. Theoretical pI estimation placed the vaccine in the basic range at 8.20, while the negative GRAVY value of −0.030 indicated its hydrophilic nature and high solubility. These characteristics suggested that the designed vaccine could exhibit enhanced interactions with water molecules [32].

Considering the MEV’s varied half-lives in mammalian, yeast, and bacterial cells (i.e., 30, 20, and 10 h, respectively), it can be inferred that the vaccine may elicit prolonged and robust immunological responses [49,118]. The 3D structure of the MEV, the initial model generated by AlphaFold v.2, underwent refinement using the GalaxyRefine server. This refinement resulted in a substantial improvement, with the residue percentage in favorable regions increasing from an initial 87.5% to 97.1%. Additionally, the Z-score, a measure of the structural quality calculated by the ProSA server, demonstrated improvement from −7.66 to −7.72. A more negative Z-score implies high structural quality for the refined 3D model of the MEV [119]. Recognizing the significance of discontinuous B-cell epitopes in humoral immune responses [120], the ElliPro server analysis highlighted that the designed MEV could induce substantial antibody production [108].

The immunological simulation outcomes provided valuable insights into the MEV’s efficacy, revealing an antibody cascade against *S. mutans* that closely mimicked typical immune responses to pathogenic infections. The study demonstrated the MEV’s ability to improve both cell-mediated and humoral immunity, with sustained elevation in B-cell (memory and plasma) and T-cell (cytotoxic and helper) counts observed up to 350 days post-immunization. While initial immunization led to an increase in IFN-ꝩ levels, a subsequent decline and scarcity of anti-inflammatory cytokines following the third dose were recorded throughout the study period. Secondary and tertiary immune responses post-vaccination were stronger than the initial responses, resulting in significant antibody quantities and efficient antigen elimination [108].

The strategic selection of multiple TLRs is crucial for enhancing the efficacy of the MEV and promoting crosstalk between innate and adaptive immunity, leading to a more robust and enduring immune response [121]. TLRs activate distinct signaling pathways toward synergistic effects in immune activation. Previous studies have demonstrated that vaccines targeting multiple TLRs induce stronger and more diverse immune responses compared to those targeting a single TLR. This multifaceted approach reduces the risk of pathogen escape mutations, a potential consequence when targeting only one TLR [122].

In particular, TLR-2 plays a pivotal role in detecting a wide range of pathogen-associated molecular patterns (PAMPs), particularly those on the surface of bacteria and viruses. This recognition is essential for initiating immune responses against various pathogens, contributing to bacterial clearance [123,124]. TLR-3 acts preventively against infections by recognizing double-stranded RNA, a common viral intermediate, and triggering antiviral immune responses [125]. During bacterial infections, TLR-4 senses toxins or lipopolysaccharides, causing pro-inflammatory reactions that aid in eliminating invasive pathogens [126]. In parallel, MHC-I molecules convey intracellular pathogen-derived peptides to cytotoxic CD8+ T lymphocytes, leading to the elimination of infected cells, while MHC-II molecules activate immune responses and coordinate pathogen clearance by presenting extracellular pathogen-derived peptides to CD4+ T cells [127]. The MEV was formulated to engage with TLR-2, TLR-3, and TLR-4, as well as MHC molecules, effectively leveraging the innate immune system to combat a diverse array of *S. mutans* strains. Low energy scores between the MEV–TLRs and MEV–MHC complexes (Table 4), as per experimental docking, suggested strong binding affinities. It confirmed the vaccine’s potential to activate key immune pathways, stability and efficacy, and long-term broad-spectrum immunity [121,122]. The stability and flexibility of MEV, when bound to different receptors, were analyzed through MD simulation and NMA study. The MD simulation, including RMSD and RMSF analysis, showed the MEV–TLR-4 (PDB: 4G8A) complex to exhibit the highest stability and lower flexibility in the structure, followed by MEV–TLR-4 (3FXI), MEV–TLR-4 and MEV–MHC-I complexes. Consistent with these findings, the NMA study demonstrated that the MEV–TLR-4 (PDB:4G8A) complex had the lowest eigenvalue among the complexes, indicating superior stability and flexibility for large-scale conformational changes. This finding suggested that conformational changes in TLR-4 could activate downstream signaling cascades, thereby triggering the subsequent immunological responses against *S. mutans* [128]. From the perspective of mass production of the designed MEV, codon optimization for *E*. *coli* strain K12 resulted in a codon adaptability index (CAI) of 0.99 and a GC content of 49.13%, suggesting an ideal setup for mass production of the protein in *E. coli* expression systems [129,130]. The *E. coli* cell culture technique is commonly employed for large-scale production of recombinant proteins [131].

The designed MEV is developed to target both dental caries and other *S. mutans*-associated infections by inducing systemic and local immune responses. However, *S. mutans* primarily reside in the oral cavity, where the innate immune response, particularly neutrophils, is the dominant defense mechanism [132]. To effectively combat *S. mutans* in this localized environment, mucosal immunization via oral or nasal routes could be optimized to enhance local immune responses. This approach would stimulate the production of secretory IgA (sIgA), a key immunoglobulin for neutralizing pathogens at mucosal surfaces [133,134]. By targeting both systemic and mucosal immunity, the MEV could provide a comprehensive defense against *S. mutans* not only in circulation but also at the site of infection, addressing both oral and systemic infections.

In future in vitro experiments, retaining the structural integrity of the multiepitope vaccine is a key challenge, as environmental factors including pH and enzymatic degradation could destabilize the epitopes. Encapsulation in nanoparticles or liposomes is a viable solution to protect the vaccine and ensure optimal antigen presentation [135,136]. Additionally, testing immune cell interactions in vitro may be complicated by the lack of a suitable co-culture system that mimics the oral mucosa. This can be addressed by using advanced 3D cell culture models or organ-on-a-chip technologies, which more accurately simulate tissue environments [137,138]. In vivo, effective mucosal delivery poses a challenge, as inducing strong immune responses, particularly secretory IgA at mucosal sites, is difficult with traditional methods. Exploring nasal or oral delivery routes could enhance these responses. Furthermore, since *S. mutans* is part of the oral microbiome, there is a risk that altering its balance could affect other beneficial bacteria. Finally, addressing potential safety concerns, including the risk of autoimmunity or inflammation, will require preclinical testing to ensure immunogenicity without adverse reactions [139]. These future studies will be critical to translate the MEV into an effective, safe therapeutic for oral infections.

## 5. Conclusions

The designed MEV holds significant potential to revolutionize oral healthcare worldwide by offering a long-term, practical solution to dental caries. The present study employed an immunoinformatic strategy to construct a chimeric multiepitope vaccine by the integration of distinct CTL, HTL, and linear B-cell epitopes generated from five antigenic proteins produced by *S. mutans*. Our computational analysis demonstrated that the MEV design could effectively prevent and control *S. mutans* infection, providing a promising foundation for future interventions. However, while these in silico results are promising, further experimental validation is crucial to assess the vaccine’s real-world safety and efficacy. As a prerequisite for the MEV to be successfully used in oral healthcare, more research may be undertaken to understand its biological context of action and any potential interactions with the human microbiome. Additionally, integrating the MEV into broader oral health strategies, such as blending it with existing preventive measures like fluoride treatments, sealants, or probiotics, could offer a comprehensive, multi-faceted approach to caries prevention. Long-term clinical research should focus on the durability of the immune response and scalability of vaccine production to ensure that MEV can be cost-effectively mass-produced and distributed globally. By aligning these computational insights with practical dental healthcare applications, the MEV could become a vital tool in reducing the global burden of dental caries.

## Figures and Tables

**Table 1:** List of selected linear B-cell epitopes predicted by the SVMTrip server.

Sl. No	Protein	Location	Peptide Sequence	Score
1.	PBP2X	190–209	PNGTFASQFIGLAQIKENKD	1
2.	PBP2b	102–121	DIKANAKKLADMVTLTESKV	1
3.	MurG	199–218	SAGAKVFNQFISDTPELTKH	1
4.	ATP-F	132–151	DLSVLLAEKIMAKNLDKTAQ	1
5.	AGPAT	226–245	AFTYLFSWFASFVWNPEKHR	1

**Table 2:** List of selected CTL epitopes predicted by NetCTL 1.2 server.

Protein	Peptide Sequence	MHC Supertype A1 Binding Affinity *	ReScale Binding Affinity	C-Terminal Cleavage Affinity	Transport Efficiency	Prediction Score
PBP2X	LSGSNDYIY	0.479	2.0338	0.4883	2.754	2.2448
	LSFAQGFAY	0.4052	1.7204	0.8636	3.029	2.0014
PBP2b	QMDTEVATF	0.7955	3.3775	0.8953	2.779	3.6508
	YTAMQLAQY	0.6555	2.783	0.8258	3.041	3.0589
	TSSPRGQIY	0.5872	2.493	0.9516	3.041	2.7878
MurG	RVDYVTEMY	0.62	2.6325	0.9614	3.079	2.9306
	SSDDNTEIK	0.3065	1.3013	0.8676	0.436	1.4532
ATP-F	GTSLGNLLI	0.2004	0.8509	0.5643	0.471	0.9591
AGPAT	WAANGNIHY	0.5014	2.1287	0.9696	2.976	2.423
	QMDTEVATF	0.2896	1.2296	0.9366	2.354	1.4877

* A1 is an MHC class I supertype that includes alleles with similar peptide-binding specificities from the HLA-A locus.

**Table 3:** List of selected HTL epitopes that were predicted to induce INF-ꝩ, TNF, and IL-10.

Protein	MHC-II Allele	Peptide Sequence	INF-ꝩ	TNF-α	IL-10
PBP2X	HLA-DRB1*01:01	TLYSASLGGPVIKVG	1.0937	-	-
PBP2b	HLA-DRB1*01:01/HLA-DRB1*01:03	TFTLISNRINLLFFL	1.05	0.47	0.654631
MurG	HLA-DRB1*01:01/HLA-DRB1*03:01	ESDLSMGLANRIAYK	0.6066	-	-
ATP-F	HLA-DRB1*03:01/HLA-DRB1*03:01	ADLSVLLAEKIMAKN	0.9561	0.46	-
AGPAT	HLA-DRB1*01:01/HLA-DRB1*04:04	VFYTYLRSLLVFLIW	1.3433	0.48	1.381912

HLA-DRB refers to an MHC class II allele from the HLA-DR locus, involved in presenting peptides to CD4+ T cells.

**Table 4:** Molecular docking results of MEV with TLRs and MHC molecules.

Receptors	PDB ID	ClusPro Energy Score (kcal/mol)	PRODIGY Binding Affinity (ΔG)	Dissociation Constant kd (M)	No. of H-bonds	MOE Binding Affinity (kcal/mol)
TLR-2	6NIG	−1114.7	−11.6	3.10 × 10^−9^	12	−70.3042
TLR-3	1ZIW	−1134.3	−20	2.10 × 10^−15^	34	−81.1515
TLR-4	4G8A	−1397.7	−20.3	1.20 × 10^−15^	31	−84.3597
TLR-4	3FXI	−1257.8	−17.2	2.10 × 10^−13^	28	−98.4945
MHC-I	1AKJ	−1068.3	−15	2.10 × 10^−12^	22	−85.4256
MHC-II	1KG0	−1128.4	−8.6	5.3 × 10^−7^	6	−62.6244

## Data Availability

Data are with the authors and will be provided on request through corresponding authors.

## Supplementary Materials

The following supporting information can be downloaded at: https://www.mdpi.com/article/10.3390/pathogens13100916/s1, Figure S1: Secondary structure of multiepitope vaccine construct; Figure S2: The tertiary structure of the MEV construct modeled by AlphaFold v.2 tools; Figure S3: The B-cell discontinuous epitopes with a score value of more than 0.70; Figure S4: Disulfide engineering of multiepitope vaccine construct; Figure S5: Molecular docking simulation of TLR-2 proteins with the MEV; Figure S6: Molecular docking simulation of MHC-II proteins with the MEV. Figure S7. Molecular dynamics simulation of MEV construct with TLR-3 and TLR-4 (4G8A); Table S1. List eighteen *S. mutans* genomes for the generation of core-genome; Table S2. A list of selected vaccine candidate proteins for the construction of a multiepitope vaccine; Table S3. Galaxy Refine server generated 5 top Models from AlphaFold’s Rank1 model of multiepitope vaccine construct; Table S4. Predicted conformational B-cell epitope residues of multiepitope vaccine construct; Supplementary File S1.
