# Supplementary material for: Immunoinformatics Design of a Multiepitope Vaccine (MEV) Targeting Streptococcus mutans: A Novel Computational Approach
Source: Pathogens. 2024 Oct 21;13(10):916. doi: 10.3390/pathogens13100916 (PMC11509883; doi:10.3390/pathogens13100916)
Supplement: Supplementary file 1 [file pathogens-13-00916-s001.zip › Supplementary file _1.pdf]

```
# Tmpdir made /var/www/services/services/NetMHCII-2.3/tmp/netMHCIIv6S2Un
# Input is in FSA format

# Peptide length 15
        NetMHCII version 2.3.

Strong binder threshold   2.00. Weak binder threshold  10.00.

--------------------------------------------------------------------------------
-----------------
Allele          pos               peptide       core Of 1-log50k(aff)
affinity(nM)    %Rank Relia     Identity Bind Level

DRB1_0101    1      GTLYSASLGGPVIKV  YSASLGGPV  3        0.8114           7.7
 1.60  0.73        PBP2X          SB
DRB1_0101    2      TLYSASLGGPVIKVG  YSASLGGPV  2        0.8065           8.1
 1.80  0.71        PBP2X          SB
DRB1_0101    3      KDVYTTLSAPIQTYL  YTTLSAPIQ  3        0.8043           8.3
 1.90  0.77        PBP2X          SB
DRB1_0101    1      SKTFTLISNRINLLF  FTLISNRIN  3        0.8792           3.7
 0.25  0.60        PBP2b          SB
DRB1_0101    2      KTFTLISNRINLLFF  FTLISNRIN  2        0.8694           4.1
 0.40  0.56        PBP2b          SB
DRB1_0101    3      QSKTFTLISNRINLL  FTLISNRIN  4        0.8692           4.1
 0.40  0.59        PBP2b          SB
DRB1_0101    4      IHIFSQMNATSVFNT  FSQMNATSV  3        0.8457           5.3
 0.70  0.85        PBP2b          SB
DRB1_0101    5      IIHIFSQMNATSVFN  FSQMNATSV  4        0.8448           5.4
 0.70  0.79        PBP2b          SB
DRB1_0101    6      KIIHIFSQMNATSVF  FSQMNATSV  5        0.8432           5.5
 0.80  0.64        PBP2b          SB
DRB1_0101    7      TFTLISNRINLLFFL  FTLISNRIN  1        0.8368           5.8
 0.90  0.52        PBP2b          SB
DRB1_0101    8      GQSKTFTLISNRINL  FTLISNRIN  5        0.8339           6.0
 1.00  0.64        PBP2b          SB
DRB1_0101    9      HIFSQMNATSVFNTA  FSQMNATSV  2        0.8298           6.3
 1.10  0.87        PBP2b          SB
DRB1_0101   10      KKIIHIFSQMNATSV  FSQMNATSV  6        0.8072           8.1
 1.80  0.45        PBP2b          SB
DRB1_0101    1      ESDLSMGLANRIAYK  LSMGLANRI  3        0.8689           4.1
 0.40  0.90        MurG           SB
DRB1_0101    2      SDLSMGLANRIAYKF  LSMGLANRI  2        0.8640           4.4
 0.40  0.89        MurG           SB
DRB1_0101    3      HESDLSMGLANRIAY  LSMGLANRI  4        0.8504           5.0
 0.60  0.90        MurG           SB
DRB1_0101    4      KGGFVSVPPVVAANL  FVSVPPVVA  3        0.8472           5.2
 0.70  0.92        MurG           SB
DRB1_0101    5      DLSMGLANRIAYKFA  LSMGLANRI  1        0.8412           5.6
 0.80  0.85        MurG           SB
DRB1_0101    6      SKGGFVSVPPVVAAN  FVSVPPVVA  4        0.8390           5.7
 0.80  0.90        MurG           SB
DRB1_0101    7      VHESDLSMGLANRIA  LSMGLANRI  5        0.8218           6.9
 1.30  0.90        MurG           SB
DRB1_0101    8      GGFVSVPPVVAANLL  FVSVPPVVA  2        0.8217           6.9
```

							1.30	0.90	MurG	SB
DRB1_0101	9	FSKGGFVSVPPVVAA	FVSVPPVVA	5	0.8202	7.0	1.30	0.89	MurG	SB
DRB1_0101	10	NRIAYKFATTMYTTF	YKFATTMYT	4	0.8181	7.2	1.40	0.81	MurG	SB
DRB1_0101	11	RIAYKFATTMYTTFE	YKFATTMYT	3	0.8158	7.3	1.50	0.84	MurG	SB
DRB1_0101	1	VADLSVLLAEKIMAK	LSVLLAEKI	3	0.8084	8.0	1.70	0.85	ATP-F	SB
DRB1_0101	2	ADLSVLLAEKIMAKN	LSVLLAEKI	2	0.8052	8.2	1.90	0.81	ATP-F	SB
DRB1_0101	1	TAKVKIMPAAYAGPR	VKIMPAAYA	3	0.8961	3.1	0.10	0.93	AGPAT	SB
DRB1_0101	2	AKVKIMPAAYAGPRS	VKIMPAAYA	2	0.8877	3.4	0.15	0.90	AGPAT	SB
DRB1_0101	3	KTAKVKIMPAAYAGP	VKIMPAAYA	4	0.8840	3.5	0.17	0.92	AGPAT	SB
DRB1_0101	4	AKTAKVKIMPAAYAG	VKIMPAAYA	5	0.8826	3.6	0.20	0.90	AGPAT	SB
DRB1_0101	5	KVKIMPAAYAGPRSI	VKIMPAAYA	1	0.8698	4.1	0.40	0.89	AGPAT	SB
DRB1_0101	6	VFYTYLRSLLVFLIW	YTYLRSLLV	2	0.8510	5.0	0.60	0.87	AGPAT	SB
DRB1_0101	7	WSLIYRLPAMVLVAI	IYRLPAMVL	3	0.8350	6.0	0.90	0.69	AGPAT	SB
DRB1_0101	8	SLIYRLPAMVLVAIV	IYRLPAMVL	2	0.8338	6.0	1.00	0.69	AGPAT	SB
DRB1_0101	9	IAKTAKVKIMPAAYA	VKIMPAAYA	6	0.8337	6.0	1.00	0.89	AGPAT	SB
DRB1_0101	10	WWSLIYRLPAMVLVA	IYRLPAMVL	4	0.8302	6.3	1.10	0.69	AGPAT	SB
DRB1_0101	11	FYTYLRSLLVFLIWA	YTYLRSLLV	1	0.8269	6.5	1.10	0.86	AGPAT	SB
DRB1_0101	12	DDNYILVAPHRTLWD	YILVAPHRT	3	0.8164	7.3	1.50	0.87	AGPAT	SB
DRB1_0101	13	EDDNYILVAPHRTLW	YILVAPHRT	4	0.8153	7.4	1.50	0.88	AGPAT	SB
DRB1_0101	14	DNYILVAPHRTLWDP	YILVAPHRT	2	0.8128	7.6	1.60	0.88	AGPAT	SB
DRB1_0101	15	SWWSLIYRLPAMVLV	IYRLPAMVL	5	0.8094	7.9	1.70	0.65	AGPAT	SB
DRB1_0103	1	SKLKNLKKIRITLGE	NLKKIRITL	4	0.3069	1806.3	0.40	0.46	PBP2X	SB
DRB1_0103	2	GSKLKNLKKIRITLG	NLKKIRITL	5	0.2977	1996.3	0.60	0.41	PBP2X	SB
DRB1_0103	3	VGSKLKNLKKIRITL	LKNLKKIRI	4	0.2919	2124.0	0.80	0.28	PBP2X	SB
DRB1_0103	1	LFTVLLLRLAQMQLY	LLRLAQMQL	5	0.2893	2184.7	0.90	0.35	PBP2b	SB
DRB1_0103	2	ALFTVLLLRLAQMQL	LFTVLLLRL	1	0.2850	2290.3	1.00	0.31	PBP2b	SB
DRB1_0103	3	FTVLLLRLAQMQLYD	LLRLAQMQL	4	0.2847	2298.2	1.10	0.38	PBP2b	SB

DRB1_0103	4	SKTFTLISNRINLLF	TFTLISNRI	2	0.2772	2491.3	1.40	0.54	PBP2b	SB
DRB1_0103	5	KTFTLISNRINLLFF	TFTLISNRI	1	0.2768	2502.2	1.50	0.53	PBP2b	SB
DRB1_0103	6	TFTLISNRINLLFFL	TFTLISNRI	0	0.2763	2516.3	1.50	0.46	PBP2b	SB
DRB1_0103	7	FTLISNRINLLFFLI	XFTLISNRI	-1	0.2758	2529.8	1.50	0.32	PBP2b	SB
DRB1_0103	8	IVALFTVLLLRLAQM	LFTVLLLRL	3	0.2742	2574.2	1.60	0.37	PBP2b	SB
DRB1_0103	9	TVLLLRLAQMQLYDA	LLRLAQMQL	3	0.2708	2669.0	1.80	0.42	PBP2b	SB
DRB1_0103	1	SDFDVIFHSIATGKL	DFDVIFHSI	1	0.2936	2085.4	0.80	0.55	MurG	SB
DRB1_0103	2	DFDVIFHSIATGKLR	DFDVIFHSI	0	0.2908	2150.6	0.80	0.52	MurG	SB
DRB1_0103	3	PVVAANLLRIPVFVH	AANLLRIPV	3	0.2906	2154.1	0.80	0.41	MurG	SB
DRB1_0103	4	EQSDFDVIFHSIATG	DFDVIFHSI	3	0.2878	2221.0	0.90	0.60	MurG	SB
DRB1_0103	5	QSDFDVIFHSIATGK	DFDVIFHSI	2	0.2870	2240.0	1.00	0.56	MurG	SB
DRB1_0103	6	SLTIMVKVRPQVLFS	IMVKVRPQV	3	0.2769	2498.9	1.50	0.29	MurG	SB
DRB1_0103	7	PPVVAANLLRIPVFV	AANLLRIPV	4	0.2769	2500.5	1.50	0.38	MurG	SB
DRB1_0103	8	QSLTIMVKVRPQVLF	LTIMVKVRP	2	0.2767	2504.9	1.50	0.26	MurG	SB
DRB1_0103	9	VVAANLLRIPVFVHE	AANLLRIPV	2	0.2726	2617.1	1.70	0.41	MurG	SB
DRB1_0103	10	IEQSDFDVIFHSIAT	DFDVIFHSI	4	0.2710	2665.4	1.80	0.58	MurG	SB
DRB1_0103	11	LQSLTIMVKVRPQVL	LTIMVKVRP	3	0.2708	2671.0	1.90	0.28	MurG	SB
DRB1_0103	1	EAVRYPVKMLRKSDR	PVKMLRKSD	5	0.3167	1625.5	0.25	0.28	AGPAT	SB
DRB1_0103	2	TEAVRYPVKMLRKSD	AVRYPVKML	2	0.3117	1715.7	0.30	0.25	AGPAT	SB
DRB1_0103	3	AVRYPVKMLRKSDRS	PVKMLRKSD	4	0.3089	1767.0	0.40	0.37	AGPAT	SB
DRB1_0103	4	GTEAVRYPVKMLRKS	GTEAVRYPV	0	0.3064	1815.8	0.40	0.28	AGPAT	SB
DRB1_0103	5	VRYPVKMLRKSDRSL	PVKMLRKSD	3	0.3023	1899.2	0.50	0.39	AGPAT	SB
DRB1_0103	6	PGTEAVRYPVKMLRK	GTEAVRYPV	1	0.2888	2198.2	0.90	0.33	AGPAT	SB
DRB1_0103	7	NPGTEAVRYPVKMLR	GTEAVRYPV	2	0.2860	2264.4	1.00	0.34	AGPAT	SB
DRB1_0103	8	LIYRLPAMVLVAIVL	IYRLPAMVL	1	0.2818	2370.3	1.20	0.22	AGPAT	SB
DRB1_0103	9	RYPVKMLRKSDRSLI	PVKMLRKSD	2	0.2761	2520.6	1.50	0.44	AGPAT	SB
DRB1_0103	10	YPVKMLRKSDRSLIM	PVKMLRKSD	1	0.2759	2525.4	1.50	0.44	AGPAT	SB

DRB1_0103	11	DGIEEVAHRIEKEFD	GIEEVAHRI	1	0.2697	2700.7	1.90	0.61	AGPAT	SB
DRB1_0301	1	KFLDYVVGDRRVPRE	VVGDRRVPR	5	0.8176	7.2	0.08	0.78	PBP2X	SB
DRB1_0301	2	LDYVVGDRRVPRENR	VVGDRRVPR	3	0.8102	7.8	0.10	0.94	PBP2X	SB
DRB1_0301	3	FLDYVVGDRRVPREN	VVGDRRVPR	4	0.8052	8.2	0.12	0.89	PBP2X	SB
DRB1_0301	4	KKFLDYVVGDRRVPR	VVGDRRVPR	6	0.8009	8.6	0.12	0.68	PBP2X	SB
DRB1_0301	5	DYVVGDRRVPRENRE	VVGDRRVPR	2	0.7742	11.5	0.25	0.94	PBP2X	SB
DRB1_0301	6	FIIIIGTDRKFGVNL	IGTDRKFGV	4	0.7358	17.4	0.60	0.56	PBP2X	SB
DRB1_0301	7	IIIIGTDRKFGVNLS	IGTDRKFGV	3	0.7224	20.2	0.70	0.62	PBP2X	SB
DRB1_0301	8	NFIIIIGTDRKFGVN	IGTDRKFGV	5	0.7194	20.8	0.70	0.55	PBP2X	SB
DRB1_0301	9	FTSVSNDGVMLEPQF	VSNDGVMLE	3	0.7149	21.9	0.80	0.95	PBP2X	SB
DRB1_0301	10	AFTSVSNDGVMLEPQ	VSNDGVMLE	4	0.7064	24.0	0.90	0.95	PBP2X	SB
DRB1_0301	11	YVVGDRRVPRENRER	VVGDRRVPR	1	0.7046	24.4	0.90	0.85	PBP2X	SB
DRB1_0301	12	TSVSNDGVMLEPQFI	VSNDGVMLE	2	0.6910	28.3	1.10	0.93	PBP2X	SB
DRB1_0301	13	RAFTSVSNDGVMLEP	VSNDGVMLE	5	0.6806	31.7	1.30	0.95	PBP2X	SB
DRB1_0301	14	VNFIIIIGTDRKFGV	IGTDRKFGV	6	0.6805	31.7	1.30	0.46	PBP2X	SB
DRB1_0301	15	KKKFLDYVVGDRRVP	LDYVVGDRR	4	0.6652	37.4	1.60	0.41	PBP2X	SB
DRB1_0301	16	IIIGTDRKFGVNLSK	IGTDRKFGV	2	0.6552	41.7	1.80	0.69	PBP2X	SB
DRB1_0301	1	SHAITRDIINLYQKM	ITRDIINLY	3	0.7109	22.8	0.90	0.90	PBP2b	SB
DRB1_0301	2	TSHAITRDIINLYQK	ITRDIINLY	4	0.7027	24.9	1.00	0.92	PBP2b	SB
DRB1_0301	3	HAITRDIINLYQKMY	ITRDIINLY	2	0.6895	28.8	1.10	0.84	PBP2b	SB
DRB1_0301	4	EGKIISDKTTAKGTK	IISDKTTAK	3	0.6752	33.6	1.40	0.94	PBP2b	SB
DRB1_0301	5	KEGKIISDKTTAKGT	IISDKTTAK	4	0.6666	36.9	1.60	0.94	PBP2b	SB
DRB1_0301	6	TTSHAITRDIINLYQ	ITRDIINLY	5	0.6621	38.7	1.70	0.89	PBP2b	SB
DRB1_0301	7	AITRDIINLYQKMYP	ITRDIINLY	1	0.6547	41.9	1.80	0.69	PBP2b	SB
DRB1_0301	1	YNLLLTDITSKSKGK	LLTDITSKS	3	0.7685	12.2	0.30	0.98	MurG	SB
DRB1_0301	2	ESDLSMGLANRIAYK	LLTDITSKS	5	0.7601	13.4	0.40	0.98	MurG	SB
DRB1_0301	3	FYNLLLTDITSKSKG	LLTDITSKS	4	0.7590	13.6				

0.40 0.98 MurG SB
DRB1_0301 4 FNQFISDTPELTKHY FISDTPELT 3 0.7238 19.9
0.70 0.88 MurG SB
DRB1_0301 5 VFNQFISDTPELTKH FISDTPELT 4 0.7184 21.0
0.70 0.91 MurG SB
DRB1_0301 6 NQFISDTPELTKHYN FISDTPELT 2 0.7066 23.9
0.90 0.87 MurG SB
DRB1_0301 7 KVFNQFISDTPELTK FISDTPELT 5 0.6904 28.5
1.10 0.91 MurG SB
DRB1_0301 8 NDFYNLLLTDITSKS LLTDITSKS 6 0.6821 31.2
1.30 0.95 MurG SB
DRB1_0301 9 SDLSMGLANRIAYKF MGLANRIAY 4 0.6638 38.0
1.60 0.83 MurG SB
DRB1_0301 10 DLSMGLANRIAYKFA MGLANRIAY 3 0.6618 38.8
1.70 0.83 MurG SB
DRB1_0301 1 LSSVKADVADLSVLL VKADVADLS 3 0.6811 31.5
1.30 0.88 ATP-F SB
DRB1_0301 2 DLSVLLAEKIMAKNL VLLAEKIMA 3 0.6761 33.3
1.40 0.50 ATP-F SB
DRB1_0301 3 LSVLLAEKIMAKNLD LLAEKIMAK 3 0.6723 34.7
1.50 0.49 ATP-F SB
DRB1_0301 4 SSVKADVADLSVLLA VKADVADLS 2 0.6604 39.4
1.70 0.81 ATP-F SB
DRB1_0301 5 ADLSVLLAEKIMAKN VLLAEKIMA 4 0.6597 39.7
1.70 0.52 ATP-F SB
DRB1_0301 6 ALSSVKADVADLSVL VKADVADLS 4 0.6581 40.4
1.70 0.90 ATP-F SB
DRB1_0301 1 PVKMLRKSDRSLIMF LRKSDRSLI 4 0.7421 16.3
0.50 0.62 AGPAT SB
DRB1_0301 2 VKMLRKSDRSLIMFP LRKSDRSLI 3 0.7283 18.9
0.60 0.64 AGPAT SB
DRB1_0301 3 KMLRKSDRSLIMFPS LRKSDRSLI 2 0.7062 24.0
0.90 0.62 AGPAT SB
DRB1_0301 4 YPVKMLRKSDRSLIM LRKSDRSLI 5 0.6929 27.7
1.10 0.63 AGPAT SB
DRB1_0301 5 GTEAVRYPVKMLRKS VRYPVKMLR 4 0.6712 35.1
1.50 0.90 AGPAT SB
DRB1_0301 6 TEAVRYPVKMLRKSD VRYPVKMLR 3 0.6665 36.9
1.60 0.92 AGPAT SB
DRB1_0301 7 EAVRYPVKMLRKSDR VRYPVKMLR 2 0.6620 38.7
1.70 0.80 AGPAT SB
DRB1_0301 8 PGTEAVRYPVKMLRK VRYPVKMLR 5 0.6492 44.5
1.90 0.89 AGPAT SB

DRB1_0404 1 TQTQMLRAFTSVSND MLRAFTSVS 4 0.7483 15.2
0.40 0.64 PBP2X SB
DRB1_0404 2 QTQMLRAFTSVSNDG MLRAFTSVS 3 0.7467 15.5
0.40 0.70 PBP2X SB
DRB1_0404 3 VTQTQMLRAFTSVSN MLRAFTSVS 5 0.7420 16.3
0.50 0.63 PBP2X SB
DRB1_0404 4 TQMLRAFTSVSNDGV MLRAFTSVS 2 0.7338 17.8
0.60 0.70 PBP2X SB
DRB1_0404 5 GNKIIKTSVSVGSNL IIKTSVSVG 3 0.6994 25.8

```
 1.20  0.72        PBP2X        SB
DRB1_0404    6      AQGFAYSSNVGMTLL  GFAYSSNVG  2        0.6978          26.3
 1.20  0.86        PBP2X        SB
DRB1_0404    7      GVTQTQMLRAFTSVS  MLRAFTSVS  6        0.6973          26.4
 1.30  0.53        PBP2X        SB
DRB1_0404    8      NKIIKTSVSVGSNLK  IIKTSVSVG  2        0.6916          28.1
 1.40  0.74        PBP2X        SB
DRB1_0404    9      QMLRAFTSVSNDGVM  MLRAFTSVS  1        0.6908          28.4
 1.40  0.69        PBP2X        SB
DRB1_0404   10      FAQGFAYSSNVGMTL  GFAYSSNVG  3        0.6874          29.4
 1.50  0.86        PBP2X        SB
DRB1_0404   11      TGNKIIKTSVSVGSN  IIKTSVSVG  4        0.6871          29.5
 1.50  0.69        PBP2X        SB
DRB1_0404   12      QGFAYSSNVGMTLLE  GFAYSSNVG  1        0.6829          30.9
 1.70  0.83        PBP2X        SB
DRB1_0404    1      AEQIAVLATSKSDLK  QIAVLATSK  2        0.7967           9.0
 0.08  0.88        PBP2b        SB
DRB1_0404    2      TAEQIAVLATSKSDL  QIAVLATSK  3        0.7883           9.9
 0.10  0.91        PBP2b        SB
DRB1_0404    3      EQIAVLATSKSDLKG  QIAVLATSK  1        0.7862          10.1
 0.12  0.88        PBP2b        SB
DRB1_0404    4      LTAEQIAVLATSKSD  QIAVLATSK  4        0.7708          11.9
 0.20  0.93        PBP2b        SB
DRB1_0404    5      DLTAEQIAVLATSKS  QIAVLATSK  5        0.7439          16.0
 0.50  0.94        PBP2b        SB
DRB1_0404    6      KKIIHIFSQMNATSV  IIHIFSQMN  2        0.7318          18.2
 0.60  0.35        PBP2b        SB
DRB1_0404    7      KIIHIFSQMNATSVF  IIHIFSQMN  1        0.7137          22.2
 0.90  0.31        PBP2b        SB
DRB1_0404    8      EKKIIHIFSQMNATS  KIIHIFSQM  2        0.7077          23.6
 1.00  0.40        PBP2b        SB
DRB1_0404    9      QIAVLATSKSDLKGI  QIAVLATSK  0        0.7031          24.8
 1.10  0.63        PBP2b        SB
DRB1_0404   10      GVYAVVLNPNTGAVL  AVVLNPNTG  3        0.6767          33.1
 1.90  0.52        PBP2b        SB
DRB1_0404    1      GWWIRMCGAFPIDRK  WIRMCGAFP  2        0.6835          30.7
 1.60  0.86        AGPAT        SB
DRB1_0404    2      VFYTYLRSLLVFLIW  FYTYLRSLL  1        0.6807          31.7
 1.70  0.56        AGPAT        SB
DRB1_0404    3      DRSLIMFPSGSRHSS  SLIMFPSGS  2        0.6807          31.7
 1.70  0.70        AGPAT        SB
```
