# Supplementary material for: Immunoinformatics Design of a Multiepitope Vaccine (MEV) Targeting Streptococcus mutans: A Novel Computational Approach
Source: Pathogens. 2024 Oct 21;13(10):916. doi: 10.3390/pathogens13100916 (PMC11509883; doi:10.3390/pathogens13100916)
Supplement: Supplementary file 1 [file pathogens-13-00916-s001.zip › Supplementary Tables.pdf]

Supplementary Tables

Table S1. List eighteen *S. mutans* genomes for the generation of core-genome

Sl. No.	Strain Name	Accession No.
1	*Streptococcus mutans* 25145	AOCB01000001
2	*Streptococcus mutans* strain LAR01	CP023477
3	*Streptococcus mutans* strain MD	CP044493
4	*Streptococcus mutans* strain NCH105	CP044219
5	*Streptococcus mutans* strain NCTC1083	LR134320
6	*Streptococcus mutans* strain P1	CP050273
7	*Streptococcus mutans* strain P6	CP050272
8	*Streptococcus mutans* strain S1	CP050271
9	*Streptococcus mutans* strain S4	CP050270
10	*Streptococcus mutans* B04Sm5	CP061071
11	*Streptococcus mutans* strain T8	CP044492
12	*Streptococcus mutans* strain UA140	CP044495
13	*Streptococcus mutans* UA159	NC_004350
14	*Streptococcus mutans* UA159-FR	CP007016
15	*Streptococcus mutans* COCC33-14	CP101986.1
16	*Streptococcus mutans* COCC33-14R	CP101985.1
17	*Streptococcus mutans* OMZ175	CP082153.1
18	*Streptococcus mutans* strain LAB761	CP033199.1

Table S2. A list of selected vaccine candidate proteins for the construction of a multiepitope vaccine

Target Name	Mol. Wt (kDa)	Vaxign-ML Score	AlleroToP	VaxiJen	AntigenPro
PBP2X	81.7	99.5	Non-Allergen	0.5762	0.960615
PBP2B	75.15	98.9	Non-Allergen	0.6292	0.965824
MurG	40.53	90.9	Non-Allergen	0.4759	0.549342
ATP-F	17.92	90.9	Non-Allergen	0.5228	0.825725
AGPAT	29.01	90.6	Non-Allergen	0.4258	0.245626

Table S3. Galaxy Refine server generated 5 top Models from AlphaFold's Rank1 model of multiepitope vaccine construct.

Model	GDT-HA	RMSD	MolProbity	Clash score	Poor rotamers	Rama favored
Initial	1	0	1.815	1.1	4.2	91.1
MODEL 1	0.958	0.396	1.256	4.9	0	98.5
MODEL 2	0.958	0.4	1.256	4.9	0.3	98.1
MODEL 3	0.9542	0.402	1.245	4.8	0	98.1
MODEL 4	0.9623	0.378	1.19	4.1	0.3	98.5
MODEL 5	0.9526	0.415	1.294	4.3	0.6	97.6

Table S4. Predicted conformational B-cell epitope residues of multiepitope vaccine construct.

No.	Residues	Number of residues	Score
1	A:M1, A:A2, A:K3, A:L4, A:S5, A:T6, A:D7, A:E8, A:L9, A:L10, A:D11, A:A12, A:F13, A:K14, A:E15, A:M16, A:T17, A:L18, A:L19, A:E20, A:L21, A:S22, A:D23, A:F24, A:V25, A:K26, A:K27, A:F28, A:E29, A:E30, A:T31, A:F32, A:V34, A:T35, A:A36, A:A37, A:A38, A:P39, A:V40, A:A41, A:V42, A:A43, A:A44, A:A45, A:G46, A:A47, A:A48, A:P49, A:A50, A:G51, A:A52, A:A53, A:V54	53	0.845
2	A:V65, A:I66, A:L67, A:E68, A:A69, A:A70, A:G71, A:D72, A:K73, A:K74, A:I75, A:G76, A:V77, A:I78, A:K79, A:V80, A:V81, A:R82, A:E83, A:I84, A:V85, A:S86, A:G87, A:L88, A:G89, A:L90, A:K91, A:E92, A:A93, A:K94, A:D95, A:L96, A:V97, A:D98, A:G99, A:A100, A:P101, A:K102, A:P103, A:L104, A:L105, A:V108, A:A109, A:E111, A:A112, A:A113, A:D114, A:E115, A:A116, A:K117, A:A118, A:K119, A:L120, A:E121, A:A122, A:A123, A:G124, A:A125, A:T126, A:V127, A:T128	61	0.779
3	A:K270, A:V271, A:G272, A:G273, A:P274, A:G275, A:P276, A:G277, A:T278, A:F279, A:T280, A:L281	12	0.776
4	A:A250, A:T251, A:F252, A:G253, A:P254, A:G255, A:P256, A:G257, A:T258, A:L259, A:Y260	11	0.726
