# Supplementary material for: Immunoinformatics Design of a Multiepitope Vaccine (MEV) Targeting Streptococcus mutans: A Novel Computational Approach
Source: Pathogens. 2024 Oct 21;13(10):916. doi: 10.3390/pathogens13100916 (PMC11509883; doi:10.3390/pathogens13100916)
Supplement: Supplementary file 1 [file pathogens-13-00916-s001.zip › Supplementary_Figures.pdf]

Supplementary Figures:

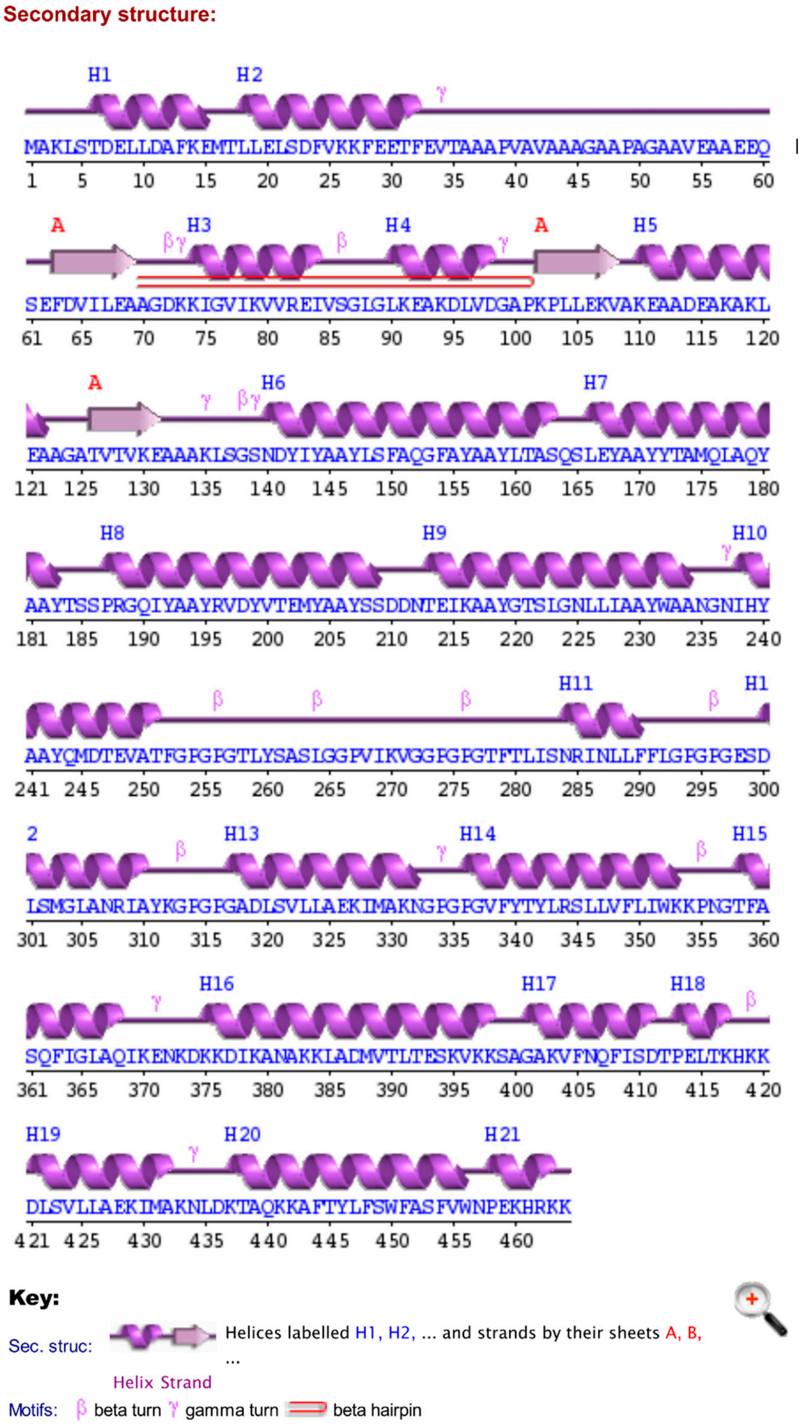

Figure S1. Secondary structure of multiepitope vaccine construct.

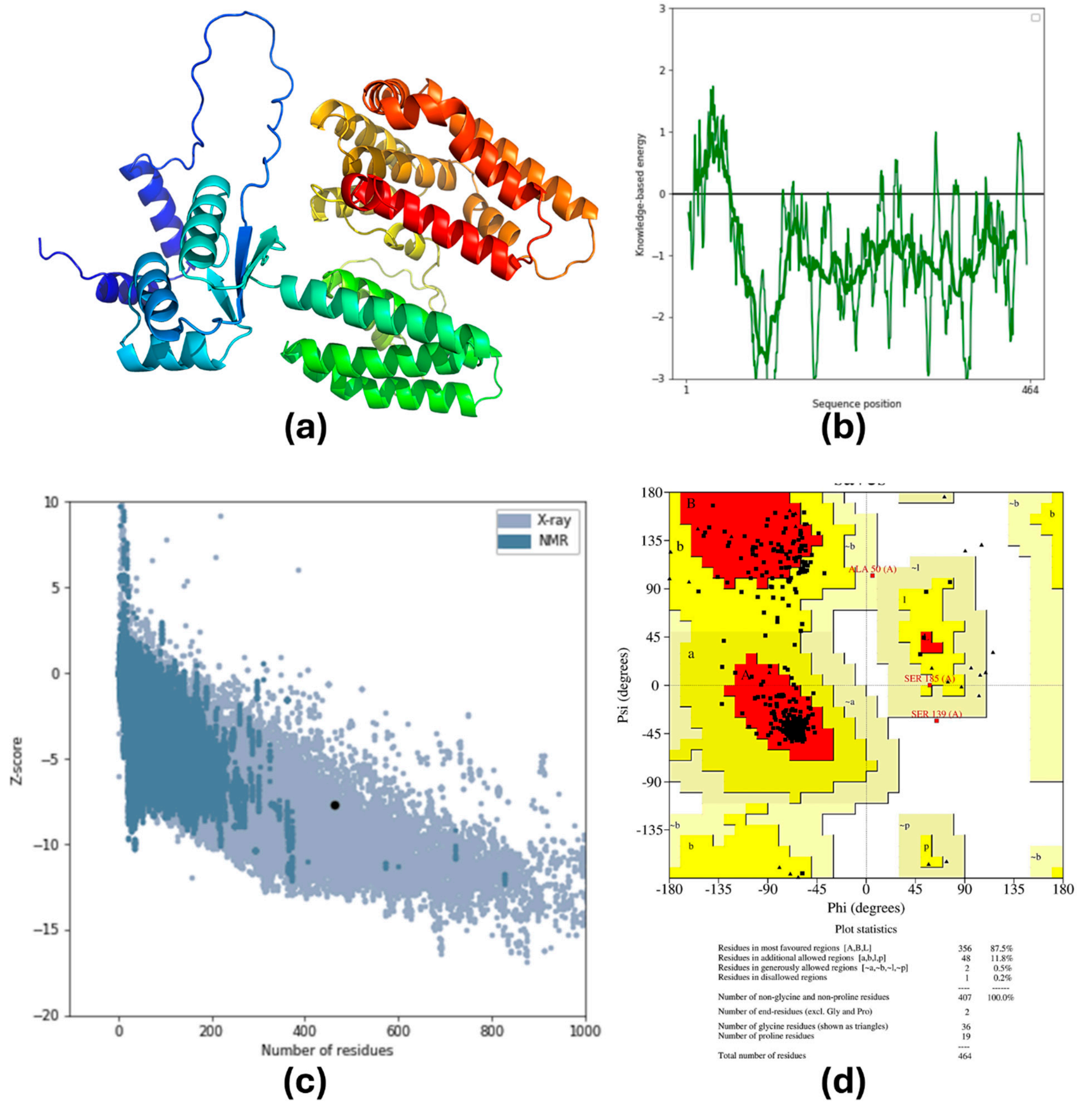

Figure S2. The tertiary structure of the MEV construct modeled by AlphaFold v.2 tools. **(a)** Initial Rank 1 structural model of MEV construct. **(b)** Energy plot model of the 3D structure of MEV. **(c)** Z-score graph via ProSA web showing the AlphaFold modeled 3D structure corresponds to X-Ray crystallographic determined structure for the protein of same sizes. **(d)** Ramachandran Plot details of the AlphaFold generated 3D model of the MEV.

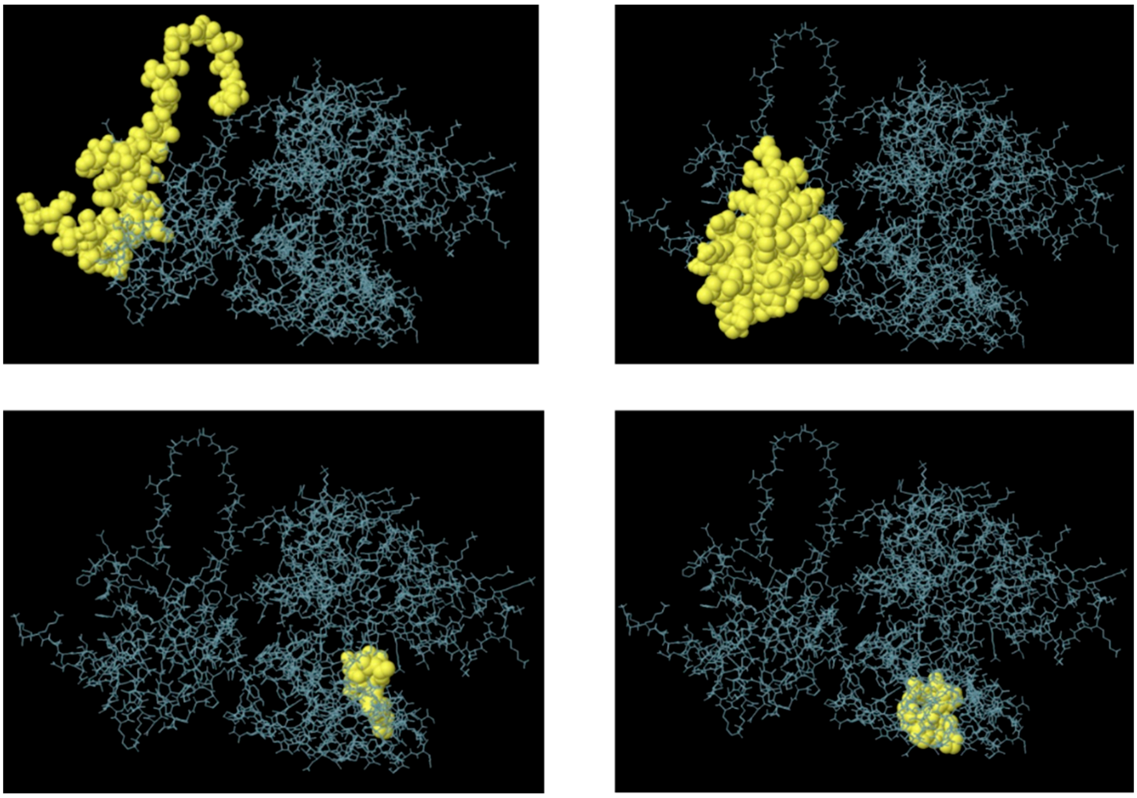

Figure S3. The B-cell discontinuous epitopes with a score value of more than 0.70.

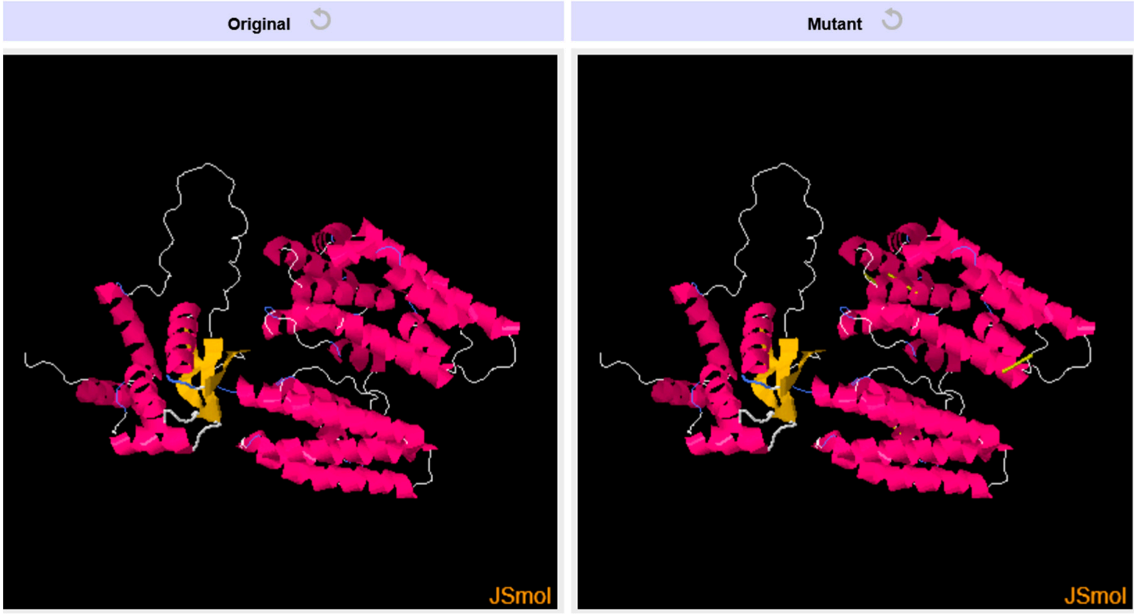

Figure S4. Disulfide engineering of multiepitope vaccine construct.

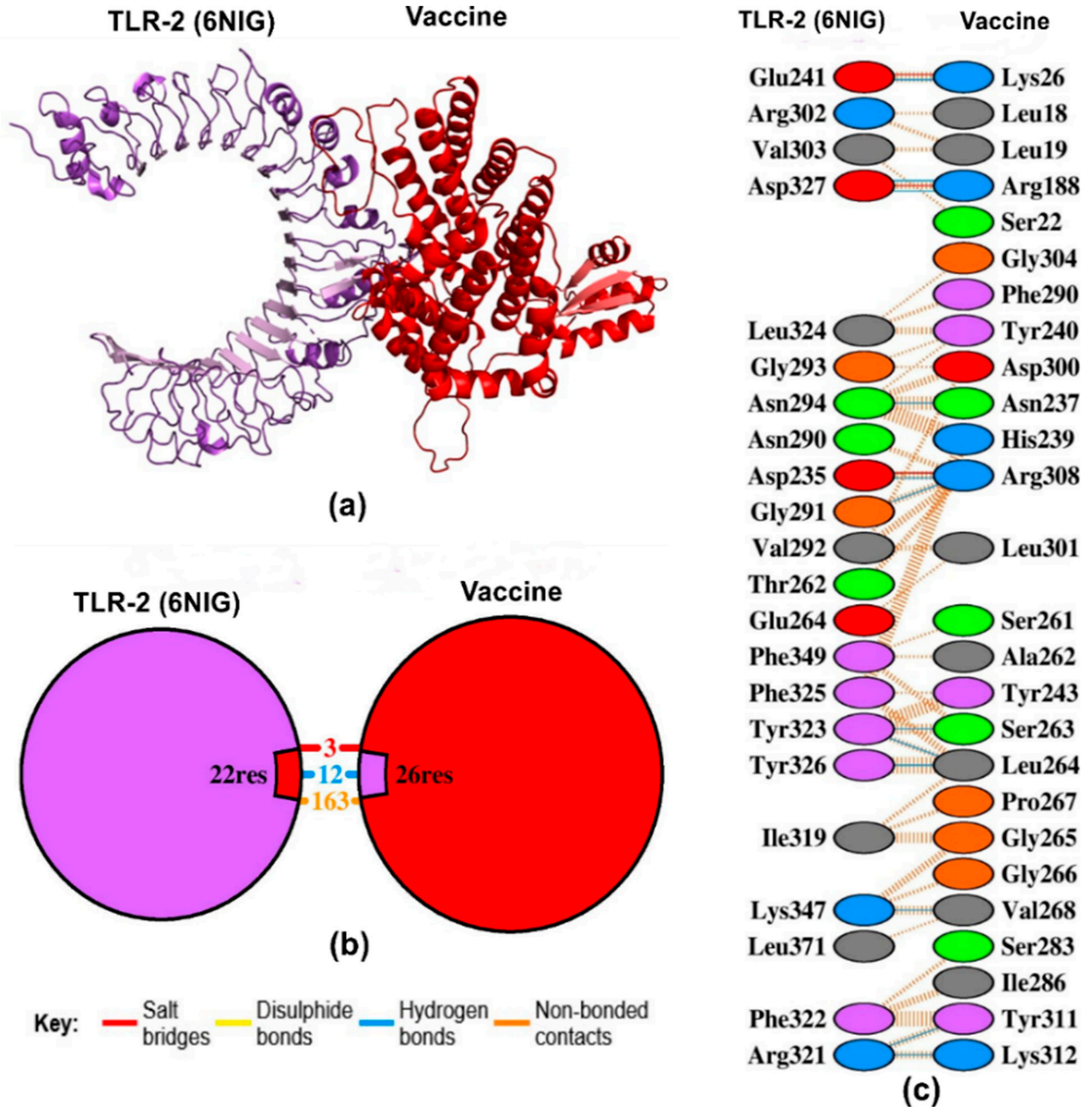

Figure S5. Molecular docking simulation of TLR-2 proteins with the MEV. (**a**) The protein-protein docking complex of TLR-2 with MEV. (**b)** The number of bonding and non-bonding contact TLR-2 -multiepitope complex. (**c**) Interacting residues of TLR-2 with the MEV, the blue line denotes H-bond formation.

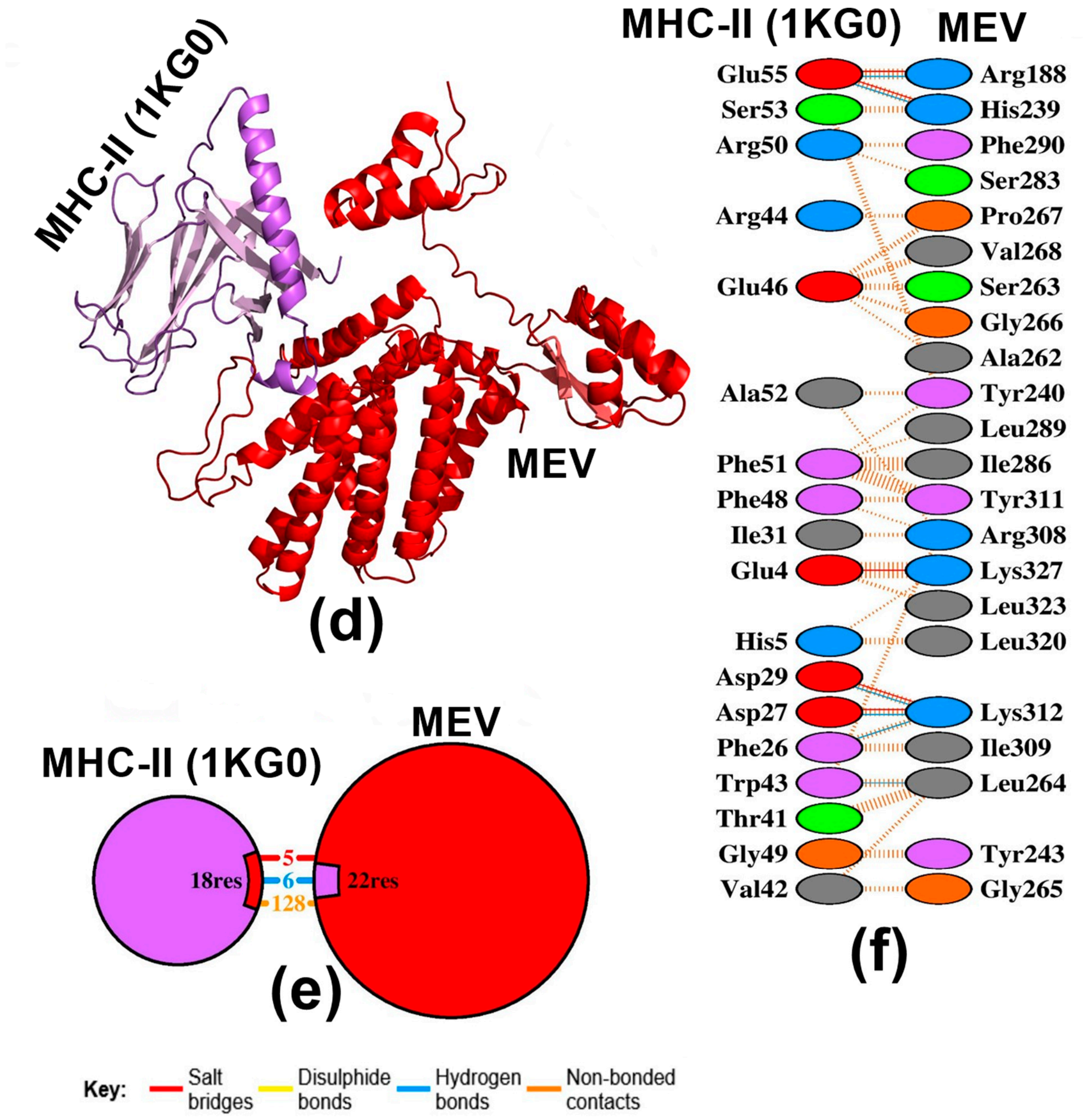

Figure S6. Molecular docking simulation of MHC-II proteins with the MEV. **(d)** The protein-protein docking complex of MHC-II (1KG0) with MEV. (**e**) The number of bonding and non-bonding contacts in MHC-II (1KG0)-MEV complex. (**f**) Interacting residues of MHC-II (1KG0) with the MEV, the blue line denotes H-bond formation.

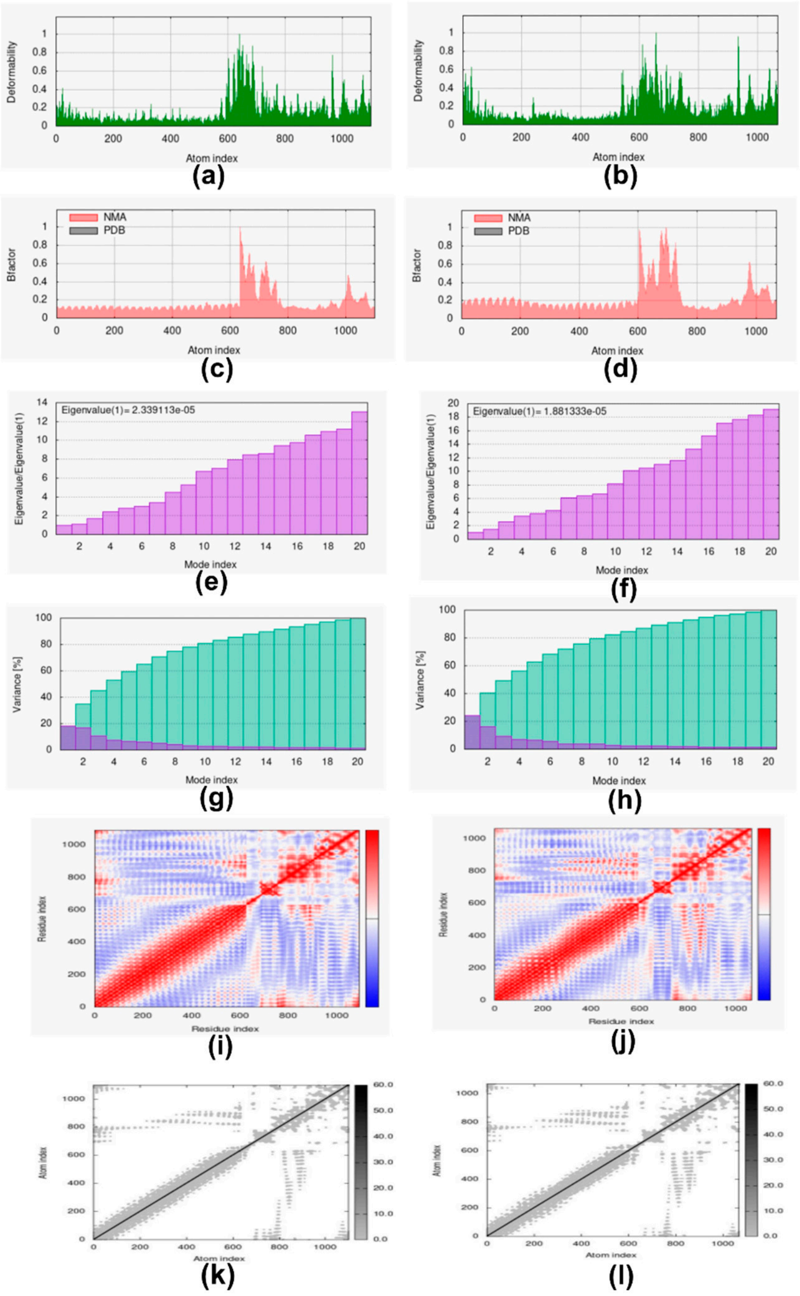

Figure S7. Molecular dynamics simulation of MEV construct with TLR-3 and TLR-4 (4G8A). (**a & b)** Deformability plots of MEV-TLR-3 complex, and MEV-TLR-4 (4G8A) complex. (**c & d)** B-factor plots of MEV-TLR-3 complex, and MEV-TLR-4 (4G8A) complex. (**e & f)** Eigenvalues plots of MEV-TLR-3 complex, and MEV-TLR-4 (4G8A) complex. (**g & h)** Variance plots of MEV-TLR-3 (1ZIW) complex, and MEV-TLR-4 (4G8A) complex; purple colors and green colors represent individual variances and cumulative variances, respectively. (**i & j)** Co-variance maps of MEV-TLR-3 complex, and MEV-TLR-4 (4G8A) complex; red, white, and blue colors represent correlated, uncorrelated, and correlated motions. Elastic network plots of MEV-TLR-3 complex, and MEV-TLR-4 (4G8A) complex, in which the dark-gray regions represent stiffer regions represented in (**j & k)**, respectively.
